# Immuno-functionomics reveals geographical variation and a role for TLR8 in mRNA vaccine responses

**DOI:** 10.1016/j.isci.2025.113839

**Published:** 2025-10-25

**Authors:** Wesley Huisman, Shohreh Azimi, Yen Nhi Nguyen, Alicia C. de Kroon, Karin de Ruiter, Dicky L. Tahapary, Mikhael D. Manurung, Cilia R. Pothast, Yvonne C.M. Kruize, Mirjam H.M. Heemskerk, Taniawati Supali, Leo G. Visser, Anna H.E. Roukens, Maria Yazdanbakhsh, Simon P. Jochems

**Affiliations:** 1 Leiden University Center for Infectious Diseases, Leiden University Medical Center, Leiden, the Netherlands; 2 Department of Internal Medicine, Faculty of Medicine, Universitas Indonesia, Jakarta, Indonesia; 3 Department of Hematology, Leiden University Medical Center, Leiden, the Netherlands; 4 Department of Parasitology, Faculty of Medicine, Universitas Indonesia, Jakarta, Indonesia

**Keywords:** Health sciences, Medicine, Medical specialty, Immunology

## Abstract

The innate immune system plays a pivotal role in pathogen defense via pattern recognition receptor sensing, initiating responses upon infection or vaccination. Understanding its functional capacity is crucial for deciphering correlates of vaccine efficacy and understanding responses to infection. Here, we developed a holistic approach to study immune function, generating >3,100 readouts across 16 cell types, 18 pattern recognition receptors, and 11 produced cytokines using spectral flow cytometry. To explore geographical variation, we compared Europeans and urban and rural Indonesians. We observed different immune responses, such as increased interleukin (IL)-1β production in rural Indonesians and impaired interferon (IFN) γ production by innate lymphocytes after Toll-like receptor (TLR) 8 stimulation. In Europeans vaccinated with mRNA-1273, baseline IFNγ production by innate lymphocytes correlated with SARS-CoV-2 spike-specific immune responses. *In vitro* mRNA vaccine stimulation also induced IFNγ production, which was TLR8 dependent and reduced in rural Indonesians. This study highlights functional immune diversity and TLR8’s potential role in mRNA vaccine responses.

## Highlights

• Spectral flow assay maps innate immune function across 16 cell types using 18 PRR sensors
• Innate responses in rural Indonesians differ from those in urban Indonesians and Europeans
• Baseline TLR8-driven capacity correlates with spike-specific mRNA-1273 responses
• *In vitro* innate lymphocyte responses to mRNA vaccines depend on TLR8 sensing

## Graphical abstract

**Figure:**
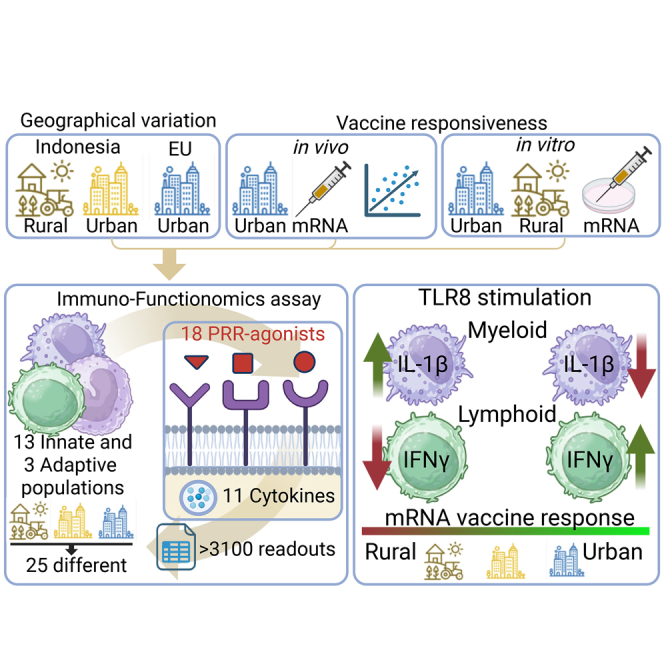

## Introduction

The innate immune system plays a critical role in protecting against infections and shaping adaptive immunity. It consists of various cell types that express unique combinations of pattern recognition receptors (PRRs) which sense pathogen-associated molecular patterns (PAMPs).^1^ These PRRs can be classified based on their location and type of PAMPs they detect. For instance, extracellular PRRs, such as Toll-like receptors (TLRs) 1, 2, 4, 5, and 6 and Dectin 1 and 2, recognize components from bacteria, parasites, and fungi. Meanwhile, endosomal and cytosolic PRRs, like TLR3, 7, 8, and 9; RIG-I; STING; and cGAS, detect nucleic acids and are essential for sensing intracellular pathogens, including viruses. When activated, PRRs initiate signaling cascades involving nuclear factor-kappa B (NF-κB) and/or interferon (IFN) regulatory factors leading to the production of IFNs and proinflammatory cytokines, which in turn shape adaptive immunity.^2^

The importance of reactivity of the innate immune system toward vaccines has already been shown before, whereby levels of activated innate cells *in vivo* early after vaccination consistently correlate with the induction of robust adaptive immunity.^3^^,^^4^ Systems biology approaches have identified potential predictive gene signatures of good universal vaccine responses,^5^^,^^6^^,^^7^^,^^8^^,^^9^ like enriched pro-inflammatory response genes downstream of NF-κB^9^ and/or T cell-enriched modules and B cell receptor signaling.^5^ However, these signatures were mainly predictive for vaccine responses in young adults and could neither be validated in cohorts of older adults nor did they include a diverse geographical variation. Indeed, predicting vaccine responses based on baseline phenotypic immunological data remains challenging, as cellular phenotype and transcriptome do not necessarily reflect functional capacity.

The individual and population-level variability in immune responses is significant, with factors contributing to this variation that are not yet fully understood. Notably, vaccine immunogenicity is often suboptimal in populations most at risk from infectious diseases, including older adults, infants, and individuals in low- and middle-income countries (LMICs). Various factors may contribute to the reduced vaccine immunogenicity observed in individuals from LMICs,^10^ such as chronic exposure to environmental pathogens, parasites, and co-infections, which can lead to an anti-inflammatory or hyporesponsive state of the immune system. This affects not only the immunogenicity of vaccines but also modulates responses to infections, such as SARS-CoV-2.^11^ Even within a country, the state of the immune system can vary,^12^ as people in rural areas of LMICs often have greater exposure to microbes and parasites than people living in urban areas.^13^^,^^14^ This is mirrored in the immunological phenotype, as, for example, by comparing the immune system of Indonesians living on rural Flores island to Indonesians living in the Indonesian capital of Jakarta, it was found that the immune system of urban Indonesians was more similar to the immune system of Europeans.^12^ However, despite the described general hyporesponsiveness and observed differences in trained immunity,^15^ the exact pathways of the innate immune system affected remain unclear.

Our study aimed to explore the functional capacity to different PRR stimulations of the innate immune system using an “immuno-functionomics” approach. We hypothesized that functional responses would vary in a cell-type and PRR-specific manner across individuals from different geographic regions and could predict vaccine responses. We found that activation of innate lymphocyte populations following TLR8 stimulation was reduced in rural Indonesians. Activation of these innate lymphocyte populations is also predictive of mRNA vaccine responses in an independent European vaccination cohort. *In vitro* responses to mRNA stimulation were reduced in rural Indonesians and could be abrogated by blocking TLR8. This highlights the potential for predicting vaccine responses based on functional capacity, allows identification of differences in capacity across regions, and reveals a previously unappreciated role for TLR8 sensing in mRNA vaccine responses.

## Results

### Simultaneous characterization of innate and adaptive immune cell functionality

**Figure 1:**
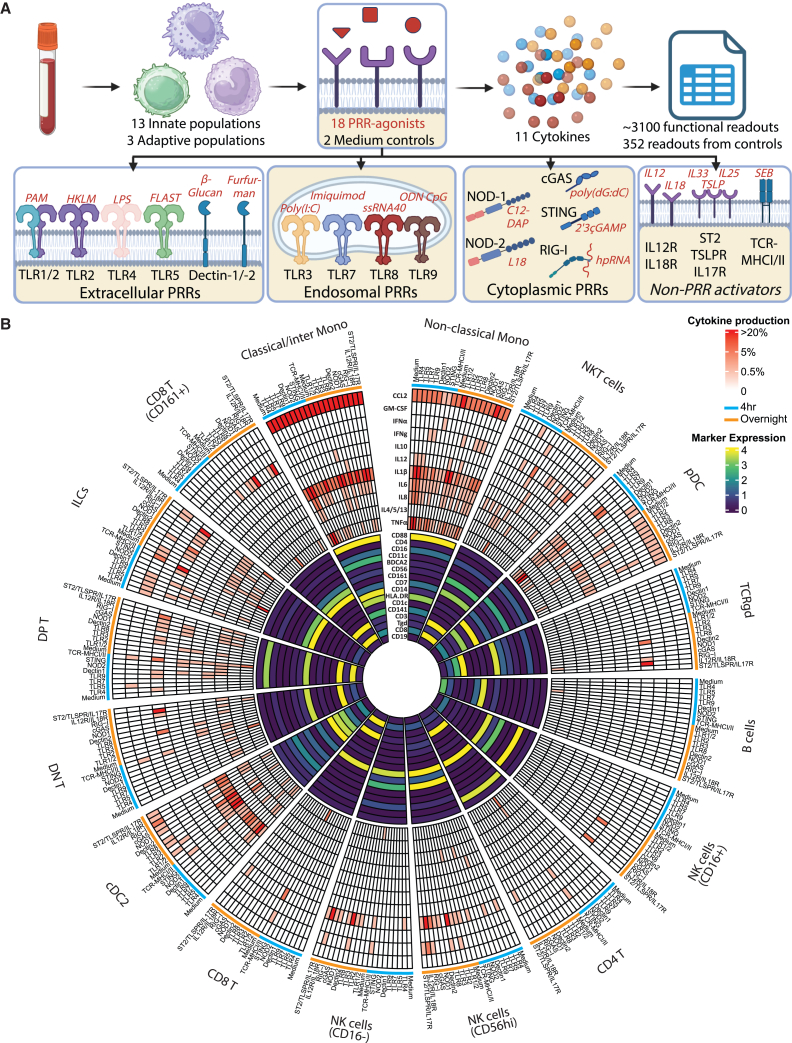
Quantification of innate and adaptive immune cell populations and their function against different PRR agonists To study the response of innate populations to stimulation with different PRR agonists, 18 PBMC samples (cohort 1) were used to generate a holistic overview of the various PRR signaling pathways across the immune system. (A) Overview of the functional assay whereby PBMCs were stimulated with 10 different PRR agonists overnight and 8 different PRR agonists for 4 h. Production of 11 different cytokines by 16 different immune cell populations was measured for each stimuli, thereby generating ∼3,100 readouts. Created in BioRender. Jochems, S. (2025) https://BioRender.com/fmavis0. (B) Heatmap of marker expression is shown in the inner circle for 16 different immune cell populations that were manually gated. Median intensity per population is shown after arcsinh transformation. For each population, the percentage of cytokine-producing cells per cytokine in response to PRR agonists stimulated for 4 h (blue) or overnight (orange) is shown in the outer circle. The average of cytokine-producing cells is shown for each population from cohort 1. In total, 462 combinations had an average cytokine production of >0.5%. mono, monocytes; cDC, classical dendritic cells; NKT cells, natural killer T cells.

In this study, we measured the production of 11 different cytokines by 16 immune cell populations in response to stimulation with 18 different stimuli, generating approximately 3,100 functional readouts per sample (Figures 1A, 1B, S1, and S2, Tables 1 and 2). Peripheral blood mononuclear cells (PBMCs) from cohort 1 (Table 3; *n* = 18) were used in the experimental design. Of these combinations, 462 showed a cytokine production of at least 0.5% relative to the parent cell population (Figure 1B and S3). All populations produced multiple cytokines in response to different PRR agonists, except for B cells and CD16^+^ NK cells (Figure 1B). B cells only produced tumor necrosis factor alpha (TNFα) in response to TLR9 stimulation, while CD16^+^ NK cells only produced IFNγ in response to stimulation with recombinant IL-12 and IL-18, or after TLR8 stimulation (Figure S4A). CD16^−^ and CD56^Hi^ NK cells produced IFNγ, TNFα, and Granulocyte-macrophage colony-stimulating factor (GM-CSF) in response to several ligands, consistent with their known increased ability to produce cytokines compared to the CD16^+^ NK cell population^16^ (Figures 1B and S4B). In line with literature, responses to some PRR agonists were more predominant for specific populations than others. For example, TLR7 is known to be mainly expressed by plasmacytoid dendritic cells (pDCs)^17^ and these cells produced large amounts of TNFα and IFNα in response to TLR7 stimulation (Figure S4C). Contrary to TLR7, TLR8 is more broadly expressed by different monocyte and DC subsets^18^ and they produced IL-1β, CCL2, TNFα, IL-6, IL-8, and IL-12 in response to TLR8 (Figure S4D). Although innate lymphoid populations (e.g., NK subsets, innate lymphoid cells [ILCs], double-negative T cells (CD4^−^CD8^−^) [DN T], and double-positive T cells (CD4^+^CD8^+^) [DP T]) do not express TLR8, they produced IFNγ in response to TLR8 stimulation (Figures 1B and S4B), which has been shown to be due to accessory cytokine (IL-12 and IL-18) release by myeloid populations.^19^^,^^20^^,^^21^ Moreover, as expected, the type of produced cytokines also varied between immune populations. For example, cDC2s were among the largest IL-12 producers, while type 2 cytokines were only produced by innate lymphoid cells and T cells (Figures S4E and S4F). Monocytes showed spontaneous release of CCL2 and IL-1β cytokines even in the absence of stimulation, as described before^22^ (Figure S5).

**Table 1:** Pattern recognition receptor agonists used to investigate the function of the innate immune system

#	PRR	Agonist	Stimulation: 4 h or O/N	Company	Catalog	Working concentration
1	TLR1/2	PAM	O/N	InvivoGen	tlrl-kit1hw	1 μg/mL
2	TLR3	Poly(I:C)	O/N	InvivoGen	tlrl-kit1hw	10 μg/mL
3	TLR4	LPS	4 h	InvivoGen	tlrl-kit1hw	5 μg/mL
4	TLR5	FLAST	4 h	InvivoGen	tlrl-kit1hw	1 μg/mL
5	TLR7	Imiquimod	4 h	InvivoGen	tlrl-kit1hw	2 μg/mL
6a	TLR9	ODN2006	4 h	InvivoGen	tlrl-2006	15 μg/mL (2 μM)
6b	TLR9	ODN2395	4 h	InvivoGen	tlrl-2395	15 μg/mL (2 μM)
7	Dectin 2	Furfurman	O/N	InvivoGen	tlrl-ffm	1 μg/mL
8	TCR-MHCI/II	SEB	4 h	Sigma-Aldrich	S4881	200 ng/mL
9a	IL-12R	IL-12	O/N	InvivoGen	rcyc-hil12	50 ng/mL
9b	IL-18R	IL-18	O/N	InvivoGen	rcyec-hil18	100 ng/mL
10a	TSLP-R	TSLP	O/N	BioLegend	582406	500 ng/mL
10b	ST2	IL-33	O/N	BioLegend	581806	500 ng/mL
10c	IL-17R	IL-25 (IL-17E)	O/N	BioLegend	598902	500 ng/mL
11	TLR8	ssRNA40	O/N	InvivoGen	tlrl-kit1hw	2 μg/mL
12	NOD2	L18 MDP	4 h	InvivoGen	tlrl-lmdp	10 μg/mL
13	TLR2	HKLM	4 h	InvivoGen	tlrl-kit1hw	10E8 cells/mL
14	STING	2′,3’cGAMP	4 h	InvivoGen	tlrl-nacga23-02	10 μg/mL
15	RIG-1	3p hpRNA^a^	O/N	InvivoGen	tlrl-hprna	3 μg/mL
16	cGAS	poly(dG:dC)^a^	O/N	InvivoGen	tlrl-pgcc	0.5 μg/mL
17	NOD1	C12 iE DAP	O/N	InvivoGen	tlrl-c12dap	1 μg/mL
18	Dectin 1	β Glucan	4 h	InvivoGen	tlrl-bgp	10 μg/mL
–	a	LycoVec	–	InvivoGen	lyec-1	–

O/N, overnight.

a LycoVec (Invivogen) was added as transfection agent during stimulation

**Table 2:** Panel of different monoclonal antibodies used for analysis of the function of immune cell populations

#	Marker	Fluorochrome	Company	Cat number	Clone	Dilution	Ref. control
1	CD88	APC	Miltenyi Biotec	130-104-286	C5AR	1/100	Cells
2	CD4	APC-Fire810	BioLegend	344661	SK3	1/400	Cells
3	CD16	BUV563	BD Biosciences	748851	3G8	1/800	Cells
4	CD11c	BUV615	BD Biosciences	752323	3.9	1/50	Cells
5	CD303	BUV661	BD Biosciences	749920	V24-785	1/100	Beads
6	CD56	BUV737	BD Biosciences	612767	NCAM16.2	1/200	Cells
7	CD161	BUV805	BD Biosciences	749221	HP-3G10	1/100	Beads
8	CD7	BV480	BD Biosciences	566161	M-T701	1/200	Beads
9	CD14	BV570	BioLegend	301831	M5E2	1/100	Beads
10	HLA-DR	BV650	BioLegend	307649	L243	1/400	Cells
11	CD1c	BV711	BioLegend	331535	L161	1/200	Cells
12	CD141	BV750	BD Biosciences	747244	1A4	1/200	Cells
13	CD3	PE-Cy5	BD Biosciences	561007	UCHT1	1/400	Cells
14	TCRgd	PerCP-Vio 700	Miltenyi Biotec	130-114-040	REA591	1/120	Cells
15	CD8	Spark Blue 550	BioLegend	344759	SK1	1/800	Beads
16	CD19	SparkNIR685	BioLegend	302269	HIB19	1/100	Cells
17	GM_CSF	APC-Vio 770	Miltenyi Biotec	130-123-430	REA1215	1/100	Cells
18	TNFα	BUV395	BD Biosciences	563996	Mab11	1/50	Beads
19	IL-8	BV510	BD Biosciences	563311	G265-8	1/200	Cells
20	IFN-γ	BV605	BioLegend	502535	4S.B3	1/200	Cells
21	IL-10	BV785	BD Biosciences	564049	JES3-9D7	1/25	Beads
22	IL-1β	FITC	BioLegend	508206	JK1B-1	1/50	Beads
23	IL4	PE	BioLegend	500808	MP4-25D2	1/100	Beads
24	IL5	PE	BioLegend	504303	TRFK5	1/100	Beads
25	IL13	PE	BioLegend	501903	JES10-5A2	1/100	Beads
26	CCL2/MCP-1	PE-Cy7	BioLegend	502613	5D3-F7	1/400	Cells
27	IFN-α	PE-Vio615	Miltenyi Biotec	130-116-875	REA1013	1/100	Beads
28	IL-12	BV421	BD Biosciences	565023	C8.6	1/200	Beads
29	IL-6	PerCP-Cy5.5	BioLegend	501117	MQ2-13A5	1/200	Cells

**Table 3:** General participant characteristics

	-	Age mean (range)	Gender male %	Helminth infected no. (total)	CD4/CD8 median ratio (range)
Cohort 1	Rural	39 (18–55)	50	5 (6)	1.8 (1.4–3.2)
	Urban	29 (19–38)	50	NA (6)	1.2 (1.0–1.9)
	European	32 (21–45)	67	NA (6)	2.1 (0.8 –5.4)
Cohort 2	Rural	23 (18–37)	50	8 (8)	1.1 (0.9 –1.8)
	Urban	22 (18–24)	50	NA (8)	1.2 (0.7 –1.5)
	European	26 (20–30)	50	NA (8)	2.3 (1.5 –3.5)
Cohort 3	European vaccinated^a^	23 (18–30)	50	NA (18)	2.3 (1.4 –4.0)
Cohort 4	Rural	20 (18–47)	50	8 (8)	0.7 (0.5 –1.3)
	European	22 (18–30)	58	NA (12)	2.2 (1.2–4.4)

NA, not analyzed.

a Only year of birth was known.

In summary, we were able to obtain, in a single assay, a holistic overview of the various PRR signaling pathways across the immune system, providing results congruent with data from literature.^17^^,^^18^^,^^19^^,^^20^^,^^21^^,^^23^^,^^24^

### Functionality of immune cell populations differ between people from different geographical areas

**Figure 2:**
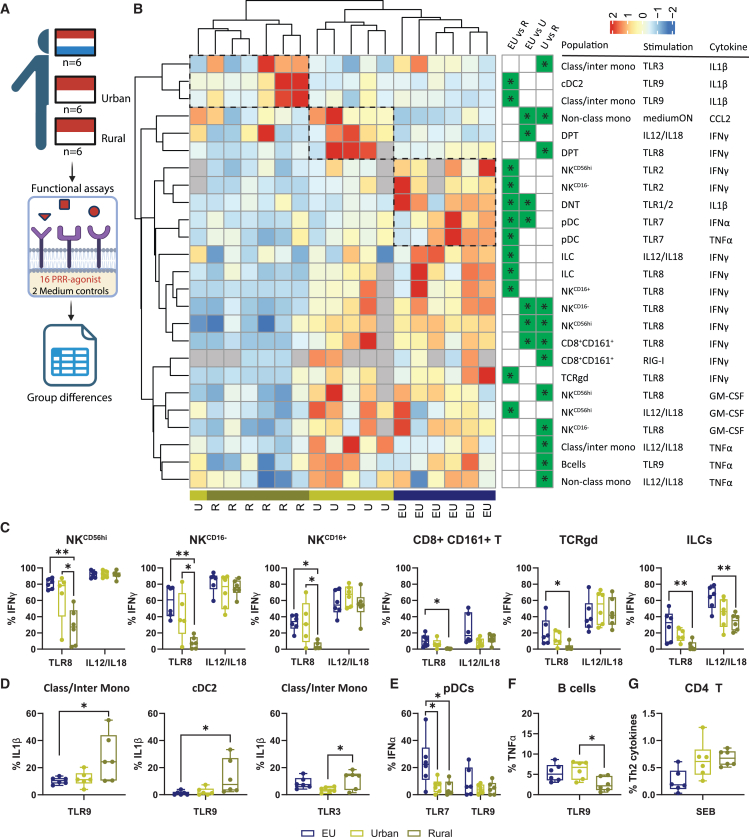
Functional differences of immune cell populations between people from different geographical areas The function of the innate immune repertoire was investigated for people from different geographical areas by using the “function-omics” assay on PBMCs from rural Indonesians (R; gold), urban Indonesians (U; yellow) and healthy Europeans (EU; blue) from cohort 1. (A) Schematic representation of the experimental setup created in BioRender. *Jochems, S. (2025)*https://BioRender.com/tmya4yp. (B) Hierarchical clustered heatmap (complete-linked, Euclidian distance) of z-scaled percentages of cytokine production for each of the 25 significant combinations (population/stimulation and produced cytokine; rows) of samples from each group (column). Gray-filled boxes indicate not performed. Dotted line represents unique clusters per group. Significant difference between groups is indicated in green-filled boxes with an asterisk. (C–G) Boxplots of immune populations that were functionally different between groups in response to different PRR agonists. Statistical differences were assessed with a Limma test with correction for testing multiple groups using Bonferroni correction. Floating boxplots depict median with minimum-maximum. ∗*p* < 0.05; ∗∗*p* < 0.01.

To compare the functional capacity of the immune system in people from rural and urban environments, we analyzed PBMCs from rural Indonesians, urban Indonesians, and healthy Europeans (*n* = 6/group, Table 3; Figure 2A). Due to limited number of PBMCs available per sample, we prioritized 16/18 stimuli and compared the 420 functional readouts that had a frequency of >0.5% cytokine-secreting cells across groups (Figure S3). Of these, 25 conditions were significantly different between the groups, with the rural Indonesians showing the most distinct profile based on hierarchical clustering (Figure 2B). Of these 25 conditions, 6 were shared between the groups. In total, each of the 13 innate populations responded differently between groups against at least one PRR agonist, with NK cells and monocytes showing most functional differences (Figures 2B and S6A).

Striking differences in function were observed between innate lymphoid populations, such as NK cell subsets, CD8^+^CD161^+^ T cells, TCRgd cells, and ILCs, which produced less IFNγ in response to TLR8 stimulation in rural Indonesians compared to urban Indonesians and Europeans (Figure 2C). The difference in IFNγ production was not due to intrinsic impairment of the innate lymphocytes, since no difference in response to IL12/IL18 was found for all these populations with the exception of ILCs. Moreover, no statistically significant differences were observed in response to other endosomal PRR agonists, although a trend for a lower response to TLR3 was observed for rural Indonesians (Figure S7). Contrary to the decreased responses of the lymphoid compartment in rural Indonesians, a more pro-inflammatory response from cDC2s and classical/intermediate monocytes was observed, with significantly more IL-1β production in response to TLR3 and TLR9 stimulation (Figures 2D and S6B). cDC2s also displayed a trend for increased IL-1β production upon TLR8 stimulation, which was negatively correlated with the frequency of IFNγ-producing lymphoid cells (Figure S8). There were also differences between urban Indonesians and Europeans, for example, pDCs from both rural and urban Indonesians produced significantly lower frequencies of IFNα and TNFα in response to TLR7 than Europeans (Figure 2E). No difference in response to TLR9 was observed from pDCs. Of the adaptive immune cell populations, B cells produced less TNFα upon TLR9 sensing in the rural Indonesian group compared to the other groups (Figure 2F). Furthermore, a non-significant trend was observed for CD4 T cells to produce more Th2 cytokines in the rural Indonesian group in response to Staphylococcus enterotoxin-B (SEB) stimulation, in line with literature^12^ (Figure 2G).

An independent cohort (Table 1; cohort 2) was used to investigate whether altered TLR levels could underlie the distinct functional responses between groups. Low/virtually absent expression of TLR 8 was found *ex vivo* for all innate lymphoid-derived cells, in line with literature.^25^^,^^26^^,^^27^ No significant differences were observed for TLR levels among innate lymphoid-derived cells, while increased TLR7, TLR8, and TLR9 expression were observed for myeloid populations of rural Indonesians, suggesting that alterations in responses were not determined by receptor abundances but likely due to alterations in downstream pathways (Figure S9).

In conclusion, a more pro-inflammatory state of myeloid immune populations, alongside increased expression of endosomal TLR sensors, was observed in rural Indonesians, while innate lymphoid populations showed decreased IFN production after TLR8 stimulation.

### Innate baseline response to TLR8 stimulation predicts immunogenicity of SARS-CoV-2 mRNA vaccination

**Figure 3:**
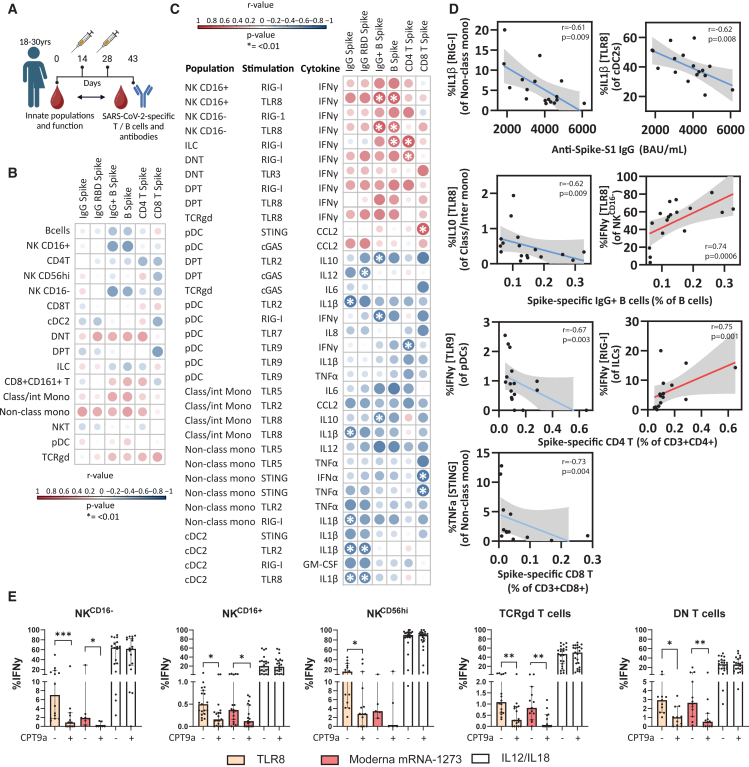
Innate baseline functional response to PRR stimulation predicts immunogenicity of SARS-CoV-2 mRNA vaccination The baseline functional capacity of PBMCs of 18 SARS-CoV-naive European individuals was measured with priority on reactivity against endosomal TLR agonists (TLRs 3, 7, 8, and 9) and cytosolic sensors cGAS, STING, and RIG-1. The functional response was then compared to the frequencies of SARS-CoV-2-specific T and B cells and titers of SARS-CoV-2-specific antibodies. (A) Overview of the study where 2 doses of Moderna mRNA-1273 vaccination were administered and peripheral blood was obtained before vaccination and 2 weeks after the second dose. (B and C) Correlation heatmap reporting Spearman correlation coefficients (r) and *p* values for each comparison. Cutoff for significant correlations are r > 0.5 and *p* < 0.01. Baseline frequencies (B) or functional responses (C) of immune cell populations are compared to frequencies of SARS-CoV-2-specific T or B cells or titers of SARS-CoV-2-specific antibodies. (D) Representative Spearman correlation plots are shown for immune cell populations where the function correlated with the vaccine immunogenicity. (E) PBMCs were stimulated with TLR8 agonist ssRNA40, Moderna mRNA1273, and IL12/IL18, with and without TLR8 inhibitor CPT9a. Correlations were assessed with multiple Spearman correlations without correction for multiple testing. Significant correlations were depicted as ∗*p* < 0.01 with r < 0.5. Statistical differences were further assessed with Wilcoxon signed-rank tests (E). ∗*p* < 0.05; ∗∗*p* < 0.01; ∗∗∗*p* < 0.001. Bar plots depict median with 95% confidence interval.

We next wanted to investigate if the functional capacity of immune cells to endosomal TLRs (TLR3, 7, 8 and 9) and cytosolic sensors (cGAS, STING, and RIG-I) could predict primary SARS-CoV-2 mRNA-1273 vaccination responses, as the underlying sensing mechanisms that engage the innate immune system for this relatively novel vaccine type are still partly unknown in humans.^28^ We measured the baseline functional capacity of PBMCs of 18 SARS-CoV-2 infection- and vaccine-naive European individuals (Table 3; cohort 3) and associated this with spike-specific antibody titers and spike-specific T and B cells^29^ 2 weeks (day 43) after the second dose of vaccination (Figures 3A and S10A). Spike-specific CD4^+^ T cells, B cells, and antibodies were all correlated with each other (Figure S10B), whereas spike-specific CD8^+^ T cells showed a distinct profile and did not correlate with the antibody production. When looking at baseline frequencies of immune cell populations, and not their functional capacity, no significant correlations (*p* < 0.01 and r > 0.5) of immune population frequencies could be found with spike-specific antibody titers or spike-specific T and B cells (Figure 3B). This highlights the complexity of linking cell frequencies to vaccination responses. However, when assessing functional capacity, significant positive correlations (*p* < 0.01 and r > 0.5) were found for different TLR8-responding NK subsets and RIG-I-responding ILCs, with the percentages of spike-specific B cells and spike-specific CD4^+^ T cells after vaccination, respectively (Figures 3C and 3D). In contrast, different subsets of IL-1β-producing monocytes and cDC2s in response to agonists for TLR2, TLR8, and RIG-I negatively correlated with the IgG^+^ spike-specific serum antibody titers detected in plasma after vaccination (Figures 3C and 3D).

The association between TLR8 responses and mRNA-1273 immunogenicity might indicate direct sensing of mRNA through this pathway, which was surprising given the replacement of uridine nucleotides with naturally occurring derivatives of uridine that serve to reduce innate sensing within mRNA vaccines.^30^ To assess whether residual TLR8 sensing of mRNA vaccines still occurs, we stimulated PBMCs *in vitro* with mRNA-1273 with and without TLR8 blocking (CPT9a). Stimulation with mRNA-1273 led to the production of IFNγ by the same innate lymphoid populations that we previously found to be predictive for a good vaccine response (CD16^+^ NK and CD16^−^ NK cells), which could be blocked by inhibiting TLR8 sensing with CPT9a (Figure 3E). As controls, we confirmed that TLR8-induced, but not IL12/18-induced, IFNγ production was reduced by TLR8 blocking. Classical/intermediate and non-classical monocytes produced IL-1β in response to mRNA-1273, but this was not affected by TLR8 blockade (Figure S11). This indicates a different sensing pathway was responsible for the inflammasome activation following mRNA-1273, likely in response to the lipid nanoparticle.^30^

In summary, high innate lymphoid-derived IFNγ responses to intracellular nucleic acid sensor stimulation, including TLR8, associated with good CD4^+^ T cell, B cell, and antibody responses to mRNA-1273 vaccination.

### *In vitro* stimulation with mRNA vaccines induces reduced IFNγ production in rural Indonesians

**Figure 4:**
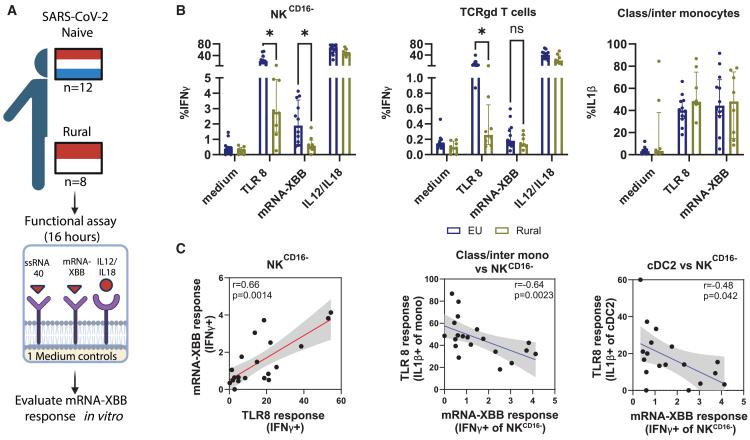
Response to SARS-CoV-2 Comirnaty mRNA-XBB vaccine *in vitro* of rural Indonesians and Europeans The response to mRNA vaccines of the innate immune repertoire from rural Indonesians (*n* = 8) and Europeans (*n* = 12) was investigated by *in vitro* stimulation with Comirnaty mRNA-XBB and was compared to the response to TLR8. (A) Schematic representation of the experimental setup created in BioRender. Jochems, S. (2025) https://BioRender.com/tmya4yp. (B) Boxplots of immune populations that were functionally different between groups in response to Comirnaty mRNA-XBB and TLR8. (C) Spearman correlation plots are shown for immune cell populations where the response to TLR8 correlated with the Comirnaty mRNA-XBB vaccine immunogenicity. Statistical differences were assessed with multiple Mann-Whitney U tests, false discovery rate corrected. ∗*p* < 0.05. Bar plots depict median with 95% confidence interval. Spearman correlation coefficients (r) and *p* values for each comparison are depicted in the correlation plots.

As we observed that TLR8 is critical for responses to mRNA vaccines, and this pathway was reduced in rural Indonesians, we hypothesized that PBMCs from rural Indonesians would show reduced responses to mRNA stimulation. Therefore, we stimulated a fourth independent cohort of PBMCs of Europeans and rural Indonesians *in vitro* with Comirnaty mRNA-XBB (mRNA-1273 was no longer available at this point; Table 3; cohort 4; Figure 4A). First, we confirmed that in this second cohort also TLR8-induced innate lymphocyte responses were reduced in rural Indonesians (Figure 4B). Moreover, IFNγ production by CD16^−^ NK innate lymphocytes upon mRNA-XBB stimulation was found to be lower in rural Indonesians compared to Europeans. Classical/intermediate monocytes also displayed a trend for increased IL-1β production upon TLR8 stimulation, which, together with cDC2s, negatively correlated with the frequency of IFNγ-producing CD16^−^ NK cells (Figures 4C and S11). IFNγ production in response to Comirnaty mRNA-XBB by CD16^−^ NK cells and TCRgd T cells correlated positively with the TLR8 response (Figure 4C).

Thus, we confirmed reduced TLR8-induced innate lymphocyte responses in an independent cohort of rural Indonesians, which correlated with reduced responses upon *in vitro* stimulation with an mRNA vaccine. Moreover, a strong inflammasome response was linked with impaired responses of innate lymphoid populations to mRNA vaccines.

## Discussion

In this study, we developed a holistic approach to study the functional capacity of the innate immune system to respond to stimulation of different PRRs. We showed that a more pro-inflammatory state of myeloid immune cells is observed in rural Indonesians. The inflammasome response of cDC2 and monocytes to TLR8 correlated negatively with the decreased function of innate lymphoid-derived cells toward mRNA vaccines, as well as decreased immunogenicity. Furthermore, we showed that IFN responses by innate lymphoid populations positively correlate with IgG+ spike-specific antibody titers and spike-specific CD4^+^ T cells after vaccination.

We showed that *in vitro* stimulation with mRNA-1273 led to IFNγ production by innate lymphoid populations in a TLR8-dependent manner. This response could be either directly mediated through upregulation of TLR8 or more likely indirectly by TLR8-induced production of IL-12/18 by myeloid cells, which in turn activates innate lymphocytes, as has been shown in several studies before.^31^ Interestingly, monocytes produced IL-1β after mRNA-1273 stimulation, but this was not mediated through TLR8. This indicates that a different sensing pathway was responsible for the inflammasome activation in these cells, potentially in response to the lipid nanoparticle.^30^ Nevertheless, our results put TLR8 sensing as crucial for the response to mRNA vaccines both *in vitro* and *ex vivo*. TLR8 was previously not identified as an important pathway in a study where mice were vaccinated with BNT162b2, as mice do not have functional TLR8.^32^ While the modification of the nucleic acids in the mRNA vaccine should prevent sensing by TLR8,^33^^,^^34^ our data indicate that this reduction is not complete in human-derived immune cells. This would have strong implications not only for mRNA-based vaccines but also other vaccines that are reportedly partially mediated through TLR8, such as the rotavirus vaccine Rotarix.^35^

Predictions for a good vaccine response often only rely on the produced antibodies. Here, we looked not only at the produced antibodies but also at the whole cellular response and memory formation of B and T cells. While antibody, B cell, and CD4^+^ T cell responses showed good correlations with each other and the functional pathways that were predictive of them, CD8^+^ T cells were not associated with them. In line with the separate mode of action of CD8^+^ T cell induction, mice infected with hookworms demonstrated compromised spike-specific T cell responses, although this did not affect antibody responses.^36^ Thus, different response pathways are associated with CD8^+^ T cell immunity compared to humoral immunity, and this increased granular understanding has implications for (1) vaccines against pathogens for which a good CD8^+^ T cell response is wanted or (2) for individuals who are not able to generate adequate antibodies.

The reasons behind vaccine hyporesponsiveness for individuals from LMICs are still poorly understood since various factors may contribute to this reduced immunogenicity.^10^ In general, few studies highlight the role of an increased pro-inflammatory transcriptomic profile, which was associated with reduced vaccine responses.^5^^,^^6^^,^^8^ Such pro-inflammatory profiles might be due to chronic (viral) pathogen exposure, potentially also highlighted by the relatively low CD4:CD8 ratio observed for our Indonesian cohorts. Additionally, the phenotype of immune cells from individuals infected with helminths is known to significantly change, showing that urban Indonesians resembled Europeans more closely.^12^ Although no information on helminth infections was available for urban individuals, reports indicate a very low incidence for helminth infections for high socioeconomic status individuals from central Jakarta.^12^^,^^37^ However, studies looking at pro-inflammatory transcriptomic profiles were neither able to pinpoint which cells displayed this pro-inflammatory profile and if that would impact specific PRR pathways nor did they investigate this in an LMIC setting. Our approach allowed us to investigate this further, where in rural Indonesians we found a strong pro-inflammatory cDC2 response toward TLR8 and TLR9, which could specifically impact the NK-dendritic cell immune axis, which is important in the initiation and coordination of adaptive immunity.^38^^,^^39^ Indeed, IL-1β production by cDC2s was negatively associated with IFNγ production by innate lymphoid populations upon TLR8 and Comirnaty mRNA-XBB stimulation. Whether this can be fully attributed to the inflammasome itself remains difficult, since it has also been reported that IFNγ is a crucial regulator of inflammasome activation.^40^^,^^41^

Higher IFNγ production upon TLR8 stimulation was associated with improved *in vivo* responses to SARS-CoV-2 in Europeans. Interestingly, several reports highlight, among others, IFNγ as a signature early after vaccination associated with effective (humoral) immune responses to BNT172b2 mRNA vaccination.^42^^,^^43^ In line with our results, we showed that IFNγ-producing DN T, DP T, ILCs, and NK cells were predictive of a good humoral response for Moderna’s mRNA-1273, allowing in-depth analysis on which cells are responsible for the production of IFNγ. Together with the reduced IFNγ responses upon TLR8 or mRNA vaccine stimulation in rural Indonesians, this raises the hypothesis that mRNA vaccines might suffer from vaccine hyporesponsiveness when deployed in LMIC. While there is no comparative *in vivo* data yet of mRNA vaccination in individuals from LMICs compared to high-income countries, a clinical trial with the mRNA-1644 HIV vaccine is underway in Rwanda and South-Africa (NCT05414786). Another vaccine for which TLR8 was found to be important, the rotavirus vaccine Rotarix,^35^ has been shown to be one of the vaccines that also suffers from decreased efficacy in LMICs.

In summary, our study reveals key insights into the interplay between innate immune system responses, geographical variation, and vaccination outcomes, highlighting the predictive potential of baseline immune parameters and the critical role of specific PRR pathways in optimizing vaccine design and addressing global disparities in immunogenicity.

### Limitations of the study

There are limitations to our study. Limited numbers of samples were available, and, therefore, we used a discovery/validation approach to validate key findings. It is known that the duration of *in vitro* stimulation impacts the cytokines that are released. Therefore, a limitation of our approach is that we are only measuring a snapshot (4 and 20 h) of cytokine production. These different stimulation durations for the PRR agonists make it furthermore difficult to compare them to each other. Also, in our approach we specifically stimulate only single PRR pathways, while it is known that stimulation of multiple different pathways simultaneously^44^ or in combination with Fcγ receptors^45^ can lead to synergistic responses. However, our parsimonious approach allows us to identify specific pathways that may be crucial in vaccine responses or highlighting differences in geographical areas. Finally, stimulating each sample with 20 different conditions requires a large enough number of cells that may not be available in all settings, for example, pediatric studies.

## Resource availability

### Lead contact

Requests for further information and resources should be directed to and will be fulfilled by the lead contact, Wesley Huisman w.huisman@lumc.nl.

### Materials availability

This study did not generate unique reagents. All materials used in this study will be made freely available upon request and the completion of applicable material transfer agreements. The information about reagents or other materials is provided in the key resources table.

### Data and code availability

• All data for this study are within the main/supplementary figures and tables. Data reported in this paper will be shared by the lead contact upon request.
• Codes used to analyze the data are available on GitHub and are listed in the key resources table.
• Any additional information required to reanalyze the data reported in this paper is available from the lead contact upon request.

## Acknowledgments

This work was supported by Leiden University Funds (LUF) awarded to S.P.J. This study was additionally supported by grants from the 10.13039/501100000781European Research Council (ERC) via the ERC Advanced Grant “REVERSE” awarded to M.Y. (grant no 101055179), The Royal Netherlands Academy of Arts and Science (KNAW), ref. 57-SPIN3-JRP and Universitas Indonesia (research grant BOPTN 2742/H2.R12/HKP.05.00/2013.), crowdfunding (Wake Up to Corona, a crowdfunding for COVID-19-related research set up by Leiden University, the Netherlands), and applications through Bontius Stichting, Leiden (BS140-20210186), U-Needle (developer and producer of the Bella-mu needle), and two Dutch philanthropic organizations (SAL Stichting Apothekers and Diraphte). The sponsors are nonprofit organizations that support science in general. They had no role in gathering, analyzing, or interpreting the data. The authors gratefully acknowledge the Flow Cytometry Core Facility (FCF) at LUMC, Leiden, The Netherlands, for technical support regarding spectral flow cytometry.

## Author contributions

Conceptualization and study design, S.P.J., M.Y., D.L.T., L.G.V., A.H.E.R., M.H.M.H., and W.H.; sample collection and processing, M.D.M., Y.C.M.K., A.C.d.K., W.H., and K.d.R.; measurements, S.A.,Y.N.N., A.C.d.K., W.H., and C.R.P.; data analysis, W.H. and S.P.J.; supervision of work, S.P.J. and M.Y.; manuscript writing, W.H. All authors read and approved the manuscript. All authors had access to the data used in the study and accept responsibility for the decision to submit the manuscript for publication.

## Declaration of interests

The authors declare no competing interests.

## STAR★Methods

### Key resources table

**Table:** 

REAGENT or RESOURCE	SOURCE	IDENTIFIER
**Antibodies**		

anti-CD88-APC	Miltenyi Biotec	Cat#130-104-286;RRID:AB_2659433
anti-CD4-APC-Fire810	Biolegend	Cat#344661;RRID:AB_2860883
anti-CD16-BUV563	BD Biosciences	Cat#748851;RRID:AB_2873254
anti-CD11c-BUV615	BD Biosciences	Cat#752323;RRID:AB_2875840
anti-CD303-BUV661	BD Biosciences	Cat#749920;RRID:AB_2874159
anti-CD56-BUV737	BD Biosciences	Cat#612767;RRID:AB_2860005
anti-CD161-BUV805	BD Biosciences	Cat#749221;RRID:AB_2873599
anti-CD7-BV480	BD Biosciences	Cat#566161;RRID:AB_2739521
anti-CD14-BV570	Biolegend	Cat#301831;RRID:AB_10897803
anti-HLA-DR-BV650	Biolegend	Cat#307649;RRID:AB_2562544
anti-CD1c-BV711	Biolegend	Cat#331535;RRID:AB_2629759
anti-CD141-BV750	BD Biosciences	Cat#747244;RRID:AB_2871963
anti-CD3-PE-Cy5	BD Biosciences	Cat#561007;RRID:AB_10584322
anti-TCRgd-PerCP-Vio 700	Miltenyi Biotec	Cat#130-114-040;RRID:AB_2733073
anti-CD8-Spark Blue 550	Biolegend	Cat#344759;RRID:AB_2819982
anti-CD19-SparkNIR685	Biolegend	Cat#302269;RRID:AB_2860769
anti-GM_CSF-APC-Vio 770	Miltenyi Biotec	Cat#130-123-430;RRID:AB_2811522
anti-TNF-a-BUV395	BD Biosciences	Cat#563996;RRID:AB_2738533
anti-IL-8-BV510	BD Biosciences	Cat#563311;RRID:AB_2738132
anti-IFN-γ-BV605	Biolegend	Cat#502535;RRID:AB_11125368
anti-IL-10-BV785	BD Biosciences	Cat#564049;RRID:AB_2738563
anti-IL-1β-FITC	Biolegend	Cat#508206;RRID:AB_345362
anti-IL4-PE	Biolegend	Cat#500808;RRID:AB_315127
anti-IL5-PE	Biolegend	Cat#504303;RRID:AB_315327
anti-IL13-PE	Biolegend	Cat#501903;RRID:AB_315198
anti-CCL2/MCP-1-PE-Cy7	Biolegend	Cat#502613;RRID:AB_2734490
anti-IFN-α-PE-Vio615	Miltenyi Biotec	Cat#130-116-875;RRID:AB_2727735
anti-IL-12-BV421	BD Biosciences	Cat#565023;RRID:AB_2739045
anti-IL-6-PerCP-Cy5.5	Biolegend	Cat#501117;RRID:AB_2572039
anti-TLR4-BV421	Biolegend	Cat#312811;RRID:AB_10896054
anti-TLR2-Pe-Cy7	Biolegend	Cat#309721;RRID:AB_2876608
anti-CD45-AF700	Biolegend	Cat#368514;RRID:AB_2566374
anti-CD16-BUV563	BD Biosciences	Cat#748851;RRID:AB_2873254
anti-CD4-cFLuor BYG750	CYTEK	Cat#SKU R7-20160
anti-TLR3-APC	R&D systems	Cat#IC1487A;RRID:AB_3654756
anti-TLR7-FITC	Biolegend	Cat#376907;RRID:AB_2922600
anti-TLR8-PE	Biolegend	Cat#395503;RRID:AB_2801048
anti-TLR9-apc-f810	Biolegend	Cat#394811;RRID:AB_2927844
anti-CD45-pacificBlue	Biolegend	Cat#368540;RRID:AB_2716030
anti-CD45-CF568 (self-conjugated)	N/A	N/A
anti-CD45-AlexaFluor700	Biolegend	Cat#368514;RRID:AB_2566374
anti-CD45-RealBlue780	BD Biosciences	Cat#568747;RRID:AB_3684512
anti-CD45-PeCy7	BD Biosciences	Cat#557748;RRID:AB_396854
Live/Dead Blue fixable Blue Dead Cell Stain	Invitrogen	Cat#L23105;RRID:AB_3717566

**Biological samples**		

Cryopreserved PBMCs from healthy adults (Europe)	LUMC Blood Bank	L19.002
Cryopreserved PBMCs from healthy adults (Indonesia)	SugarSPIN trial	doi.org/10.1186/ISRCTN75636394
Cryopreserved PBMCs from vaccinated healthy adults (Europe)	IDSCOVA trial	EUCTR2021-000454-26-NL

**Chemicals, peptides, and recombinant proteins**		

PAM	InvivoGen	Cat#tlrl-kit1hw
Poly(I:C)	InvivoGen	Cat#tlrl-kit1hw
LPS	InvivoGen	Cat#tlrl-kit1hw
FLAST	InvivoGen	Cat#tlrl-kit1hw
Imiquimod	InvivoGen	Cat#tlrl-kit1hw
ODN2006	InvivoGen	Cat#tlrl-2006
ODN2395	InvivoGen	Cat#tlrl-2395
Furfurman	InvivoGen	Cat#tlrl-ffm
SEB	Sigma-Aldrich	Cat#S4881
IL-12	InvivoGen	Cat#rcyc-hil12
IL-18	InvivoGen	Cat#rcyec-hil18
TSLP	Biolegend	Cat#582406
IL-33	Biolegend	Cat#581806
IL-25 (IL-17E)	Biolegend	Cat#598902
ssRNA40	InvivoGen	Cat#tlrl-kit1hw
L18 MDP	InvivoGen	Cat#tlrl-lmdp
HKLM	InvivoGen	Cat#tlrl-kit1hw
2′,3’cGAMP	InvivoGen	Cat#tlrl-nacga23-02
3p hpRNA∗	InvivoGen	Cat#tlrl-hprna
poly(dG:dC)∗	InvivoGen	Cat#tlrl-pgcc
C12 iE DAP	InvivoGen	Cat#tlrl-c12dap
β Glucan	InvivoGen	Cat#tlrl-bgp
∗LycoVec ™	InvivoGen	Cat#lyec-1
Phosphate-buffered saline (PBS)	Frensium Kabi	Cat#M090001/03
UltraPure Ethylenediaminetetraacetic acid (EDTA)	Invitrogen	Cat#15575020
Fetal Calf Serum (FCS)	PAN Biotech	Cat#P30-1302
Bovine Serum Albumin Fraction V (BSA)	Roche	Cat#10735086001
RPMI 1640 medium, HEPES, no glutamine	Gibco	Cat#42401042
Penicillin/Streptomycin	Gibco	Cat#15140122
L-glutamin	Merck	Cat#G8540
Brefeldin A	Invitrogen	Cat#B7450
Brilliant Stain Buffer Plus	BD Horizon	Cat#566385
True-Stain Monocyte Blocker	Biolegend	Cat#426102
Mix-n-Stain™ CF® Dye Antibody Labeling Kit	Biotium	Cat#92235
anti-human Fc Receptor (FcR) Binding Inhibitor	Invitrogen	Cat# 14-9161-73
formaldehyde solution	Thermo Scientific	Cat#28906
BD Perm/Wash buffer	BD Biosciences	Cat#554723
AbC™ Total Antibody Compensation Beads	Invitrogen	Cat#A10497
Moderna mRNA-1273 vaccine (SARS-CoV-2)	Moderna	N/A
Comirnaty Omicron mRNA-XBB vaccine (SARS-CoV-2)	BioNTech/Pfizer	N/A
CU-CPT9a	Invitrogen	Cat# HY-112667

**Software and algorithms**		

Flowjo software Version 10	N/A	N/A
SpectroFlo Version 3	N/A	N/A
R studio Version 4.32	N/A	https://github.com/spjochems/functionomics
Prism Version 10	Graphpad	N/A

**Other**		

5-laser Aurora Cytometer	Cytek	NA

### Experimental model and study participant details

For all human individuals and study sites (Table 3), PBMCs were isolated by a standardized operating procedure across sites and cohorts using Ficoll-Isopaque separation and stored in the vapor phase of liquid nitrogen. All participants provided written informed consent before sampling according to the Declaration of Helsinki. Rural Indonesian samples were obtained as part of the SugarSPIN trial (ISRCTN; doi.org/10.1186/ISRCTN75636394), a household-based cluster-randomized double-blind trial on investigating the association between whole-body insulin sensitivity and helminth infections, that was conducted in three rural villages in Nangapanda, Ende district of Flores Island (East Nusa Tenggara), Indonesia.^46^ This study has been approved by the ethical committee of Faculty of Medicine Universitas Indonesia (ref: 549/H2.F1/ETIK/2013). For this analysis, baseline samples from 2014 (pre-COVID19) prior to any potential albendazole treatment were included.

Age- and sex-matched healthy volunteer samples pre-COVID19, consisting of Caucasians from the Netherlands and individuals from urban centres in Indonesia, such as central Jakarta, were obtained from the LUMC Blood Bank (L19.002) or the LUMC Biobank for Infectious Diseases and included in this study.

We also included samples from Dutch recipients of Moderna mRNA-1273 vaccine who participated in a study where the immunogenicity of the intradermal route administration was compared to intramuscular injection^29^ (IDSCOVA trial; EUCTR2021-000454-26-NL). In this trial, healthy adults between 18 and 30 years without laboratory-confirmed or self-reported SARS-CoV-2 infection were recruited. Only samples from SARS-CoV-2 naïve individuals without SARS-CoV-2 specific antibodies, T and B cells at baseline were included in this analysis. We did not determine the influence of gender and age in our experiments, but made sure all cohorts were balanced and matched for gender and age.

### Method details

#### *In vitro* immuno-functionomics stimulations using 18 different PRR-agonists

PBMCs were thawed in a 37°C water bath and transferred to a 15 mL centrifuge tube (Greiner, Germany) and washed by slowly adding 1 mL pre-warmed thawing medium (Roswell Park Memorial Institute (RPMI) 1640 Medium (Gibco, United Kingdom), 20% heat-inactivated fetal bovine serum (FBS) (PAN Biotech, United Kingdom), 100 μg/mL streptomycin (Gibco, United Kingdom) and 100 U/mL penicillin (Gibco, United Kingdom) (Pen/Strep). Another 4 mL of pre-warmed thawing medium was slowly added to the cell suspension. After cells were spun down 5 minutes at 450g, another 5 mL of pre-warmed thawing medium was added and cells were resuspended. After another centrifugation and removal of supernatant, cells were resuspended in RPMI medium supplemented with 10% FBS, Pen/Strep and 2.7 mM L-Glutamine (Merck, Germany) (culture medium) at 5 million cells/mL. Then, 500.000 cells (100ul in culture medium) were plated per well in 2 round bottom 96 well Nunclon™ Delta Surface plates (Thermo Fisher Scientific, Denmark). Containing in total up to 18-PRR agonists and 2x medium controls that were stimulated either overnight or for 4 hours (Table 1). For the overnight stimulation plate, cells were first rested 2-4 hours at 37°C, 5% CO_2_, before 100ul of 10 PRR-agonists or medium control was added. After overnight stimulation (approximately 16 hours), 10 μg/mL Brefeldin A (Invitrogen, USA) was added, and incubated for another 4 hours. For the 4 hour stimulation plate, cells were rested overnight at 37°C, 5% CO_2_ and the following day 8 PRR-agonists or medium control was added and incubated for 1 hour. Brefeldin A was then added and incubated for another 3 hours at 37°C, 5% CO_2_. If less than 10 million cells were recovered from a thawed vial, the stimulations to use were prioritized in accordance with the order of Table 1.

#### Flow cytometry staining

Following incubation with Brefeldin A, PBMCs were detached from the wells using 2 mM UltraPure™ EDTA, pH 8.0 (Invitrogen, USA) for 10 minutes at room temperature (RT). Cells were pelleted for 5 minutes, at 450xg at RT. For each individual stimulation, PBMCs were barcoded with 50 μL of a combination of self-conjugated CD45-CF568 (Biolegend, USA. Cat.368502) using Mix-n-Stain™ CF® Dye Antibody Labeling Kit (Biotium, Cat.92235), CD45-PacificBlue (Biolegend, Cat.368540) and/or CD45-AlexaFluor700 (Biolegend, Cat.368514) for 15 minutes at RT, followed by washing with FACS buffer (Phosphate buffered saline (PBS) containing 2 % (v/v) BSA, 2 mM EDTA) (Figure S1A). Cells were washed with sterile PBS (Fresenius Kabi, Germany) twice and pooled per plate in a 15 mL falcon tube. After centrifuging and removing supernatant, cells were resuspended and stained in 50 μl for 15 min at RT with Live/Dead Blue fixable Blue Dead Cell Stain (Invitrogen, USA) solution at 1:500 dilution mixed with anti-human Fc Receptor (FcR) Binding Inhibitor (Invitrogen, USA) and True-Stain Monocyte Blocker (Biolegend) at 1:50 dilution. Then extracellular staining was performed by adding 50 μl of 2x concentrated mix containing 16 monoclonal antibodies directed against extracellular proteins (Table 2; #1-#16) prepared in FACS buffer with 5 μl BD Horizon™ Brilliant Stain Buffer Plus (BD Biosciences, USA) for 15 min at RT. After two washes with 80 μl and 180 μl of FACS buffer, cells were fixed with 180 μl of 4% final concentration formaldehyde solution (w/v), methanol free (Thermo Scientific, USA) for 20 minutes at 4°C. Before staining with an intracellular antibody cocktail, cells were washed with 180 μl 1xFACS buffer and fixed cells were centrifuged at 800g for all subsequent steps. Cells were permeabilized in 180 μl of 1x BD Perm/Wash buffer (BD Biosciences, USA) for 15 minutes at RT and then pelleted by centrifugation. Finally, intracellular staining was performed in 100 μl for 30 minutes at 4°C with a mix containing 13 different monoclonal antibodies directed against cytokines/interleukins prepared in 1x BD Perm/Wash buffer with 10 μl BD Horizon™ Brilliant Stain Buffer Plus (Table 2; #17-#29). Monoclonal antibodies directed against Th2 cytokines IL4, IL5 and IL13 were all conjugated with PE and were measured as 1 output (Th2 cytokines). After intracellular staining, cells were washed twice with Perm/Wash buffer and finally resuspended in 400 μl FACS buffer for acquisition. Cells were acquired with fluidics boost on a 5-laser Aurora Cytometer (Cytek Biosciences, Inc, USA). Reference controls were made using either AbC™ Total Antibody Compensation Beads (Invitrogen, USA) or PBMCs (Table 2). An additional unstained control, gated on monocytes (FSC/SSC) was used for unmixing.

For expression of different TLRs, 1 million PMBCs of individuals from cohort 2 were thawed similarly as before. Cells were resuspended and stained in 50 μl for 15 min at RT with Live/Dead Blue fixable Blue Dead Cell Stain (Invitrogen, USA) solution at 1:500 dilution mixed with anti-human Fc Receptor (FcR) Binding Inhibitor (Invitrogen, USA) and True-Stain Monocyte Blocker (Biolegend) at 1:50 dilution. Extracellular staining (Table S1; #1-#16) and intracellular staining (Table S1; #17-#20) were then similarly conducted as described above. Cells were acquired with fluidics boost on a 5-laser Aurora Cytometer (Cytek). Reference controls were made using either AbC™ Total Antibody Compensation Beads (Invitrogen, USA) or PBMCs (key resources table). An additional unstained control, gated on monocytes (FSC/SSC) was used for unmixing.

#### Flow cytometry gating and analysis

After acquisition, stimulations were manually debarcoded using FlowJo software (BD) and conditions were exported as separate .fcs files according to the barcoding scheme (Figures S1A and S1B). Additionally, immune cell populations (13 innate and 3 adaptive) were manually gated and exported as .fcs files using the gating shown in Figure S2. The cDC1 subset was not exported due to the low number of cells (cut off <50 cells) that could be analyzed. The exported .fcs files of the different barcodes and populations were then combined in R resulting in 320 (16 populations x 20 stimuli) conditions for each sample. Cytokine levels were arcsinh transformed prior to gating. TNFα, CCL2 and IL1β were transformed with co-factor 1500 while IL12, IFNγ, IL10, IL6, IFNα and GM-CSF were transformed with co-factor 5000. IL8 and Th2 cytokines were transformed with cofactor 15.000. Cytokines were then gated in R per samples using a semi-automated process to obtain frequencies of cytokine producing cells for each condition and cell type. For each sample, cytokine and population, the 95% quantile in the medium control of the overnight or 4-hour stimulation was determined. This value multiplied by a factor 2, 2.5 (IL12 and type 2 cytokines) or 2.7 (IL6) to set a cutoff above which cells were defined as being cytokine positive. For IL8 and IL12, an upper arcsinh transformed limit of cut-off of 2 was further defined, and for IFNα the threshold was manually set at 2 regardless of the population. As IL1β and CCL2 were spontaneously produced by certain immune populations, the threshold for these cytokines was defined using the CD4^+^ T cell population, since they do not produce IL1β or CCL2 and had similar background fluorochrome intensity across populations, and applied to all populations. Thresholds and producing cytokines were visualized for all samples and combinations of cytokines, populations and stimulations to verify correct thresholding. For analysis of meaningful cytokine-producing cells, an average 0.5% producing cells per population and cytokine was selected as cut-off for cohorts 1 and 3. The lower limit of inclusion for this 0.5% was a median of 22 cytokine producing cells (IQR 10-66) across all populations from cohort 1 (Figure S3). All R scripts used for combining exported .fcs files, transformation and semi-automated gating are available on github (https://github.com/spjochems/functionomics).

#### *In vitro* stimulations with moderna mRNA-1273 and Comirnaty Omicron mRNA-XBB 1.5

Stimulations with mRNA-1273 (Moderna, USA) and Comirnaty Omicron mRNA-XBB 1.5 (BioNTech/Pfizer, USA) were similarly conducted as for the 10 PRR-agonists that were used overnight. The mRNA-1273 vaccine was dissolved in 500 μl of the aqueous buffer that comes with the vaccine (200ug/ml). The Comirnaty Omicron mRNA-XBB 1.5, was pre-dissolved in 3000 μl of the aqueous buffer (100 μg/ml). Stimulations were performed by adding 100 μl of the dissolved mRNA-1273 or mRNA-XBB to the wells from the 96 well plate containing cells. For stimulations where the TLR8 inhibitor CU-CPT9a (Invivogen, USA) was added, cells were prior to stimulation pre-incubated with 10 μM of CU-CPT9a for 3 hours at 37°C. Vaccines and controls were added without washing, resulting in a 5 μM CU-CPT9a concentration during the overnight stimulation. Stimulations were then barcoded similarly as before using CD45-PacificBlue (Biolegend, Cat.368540), CD45-Realblue 780 (BD, Cat. 568747), CD45-Pe-cy7 (BD, Cat. 557748) and/or CD45-AlexaFluor700 (Biolegend, Cat.368514) for 15 minutes at RT.

### Quantification and statistical analysis

Results were analyzed in R version 4.32 and Prism Version 10 (GraphPad, San Diego, CA). Statistical differences between rural Indonesians, urban Indonesians and Europeans were compared using the Limma package (v3.54.2) for all conditions that were present in at least 3 individuals per group with an average frequency >0.5% (Figure S3). Correlations between the baseline functional responses and adaptive immunity following mRNA vaccination were calculated using spearman correlation (Hmisc package v.4.8.0). Bonferroni corrections were used for the comparison of multiple groups. Heatmaps were created with the pheatmap package (v1.0.12). Further statistical details of experiments can be found in the figure legends, including the statistical tests used, exact value of n and what n represents, and whether means or medians are shown.
