# Supplementary material for: Immuno-functionomics reveals geographical variation and a role for TLR8 in mRNA vaccine responses
Source: iScience. 2025 Oct 25;28(11):113839. doi: 10.1016/j.isci.2025.113839 (PMC12651802; doi:10.1016/j.isci.2025.113839)
Supplement: Document S1. Figures S1–S12 and Table S1 [file mmc1.pdf]

iScience, Volume *28*

Supplemental information

Immuno-functionomics reveals geographical variation and a role for TLR8 in mRNA vaccine responses

Wesley Huisman, Shohreh Azimi, Yen Nhi Nguyen, Alicia C. de Kroon, Karin de Ruiter, Dicky L. Tahapary, Mikhael D. Manurung, Cilia R. Pothast, Yvonne C.M. Kruize, Mirjam H.M. Heemskerk, Taniawati Supali, Leo G. Visser, Anna H.E. Roukens, Maria Yazdanbakhsh, and Simon P. Jochems

Supplemental information

Immuno-functionomics reveals geographical variation and a role for TLR8 in mRNA vaccine responses

Wesley Huisman[1], Shohreh. Azimi[1], Yen Nhi. Nguyen[1], Alicia C. de Kroon[1], Karin de Ruiter[1], Dicky L. Tahapary[2], Mikhael D. Manurung[1], Cilia R. Pothast[3], Yvonne C.M. Kruize[1], Mirjam H.M. Heemskerk[3], Taniawati Supali[4], Leo G. Visser[1], Anna H.E. Roukens[1], Maria Yazdanbakhsh[1], Simon P. Jochems[1]

[1]Leiden University Center for Infectious Diseases, Leiden University Medical Center, The Netherlands
[2]Department of Internal Medicine, Faculty of Medicine, Universitas Indonesia, Jakarta, Indonesia.
[3]Department of Hematology, Leiden University Medical Center, The Netherlands
[4]Department of Parasitology, Faculty of Medicine, Universitas Indonesia, Jakarta, Indonesia

Correspondence to: w.huisman@lumc.nl; s.p.jochems@lumc.nl

Lead Contact: w.huisman@lumc.nl

Supplementary Table 1. Panel of different monoclonal antibodies used for analysis of the TLR expression of immune cell populations

#	Marker	Fluorochrome	Company	Cat number	Clone	Dilution	ref control
1	TLR4	BV421	Biolegend	312811	HTA125	1/25	beads
2	TLR2	Pe-Cy7	Biolegend	309721	TL2.1	1/50	beads
3	CD11c	BUV615	BD Biosciences	752323	3.9	1/50	cells
4	CD14	BV570	Biolegend	301831	M5E2	1/100	beads
5	CD303	BUV661	BD Biosciences	749920	V24-785	1/100	beads
6	TCRgd	PerCP-Vio 700	Miltenyi Biotec	130-114-040	REA591	1/120	cells
7	CD56	BUV737	BD Biosciences	612767	NCAM16.2	1/200	cells
8	CD7	BV480	BD Biosciences	566161	M-T701	1/200	beads
9	CD1c	BV711	Biolegend	331535	L161	1/200	cells
10	CD141	BV750	BD Biosciences	747244	1A4	1/200	cells
11	CD45	AF700	Biolegend	368514	2D1	1/200	cells
12	CD3	PE-Cy5	BD Biosciences	561007	UCHT1	1/400	cells
13	HLA-DR	BV650	Biolegend	307649	L243	1/400	cells
14	CD16	BUV563	BD Biosciences	748851	3G8	1/800	cells
15	CD8	Spark Blue 550	Biolegend	344759	SK1	1/800	beads
16	CD4	cFLuor BYG750	CYTEK	SKU R7-20160	SK3	1/2000	cells
17	TLR3	APC	R&D systems	IC1487A	poly	1/50	beads
18	TLR7	FITC	Biolegend	376907	S18024F	1/50	beads
19	TLR8	PE	Biolegend	395503	S16018A	1/50	beads
20	TLR9	apc-f810	Biolegend	394811	S16013D	1/50	beads

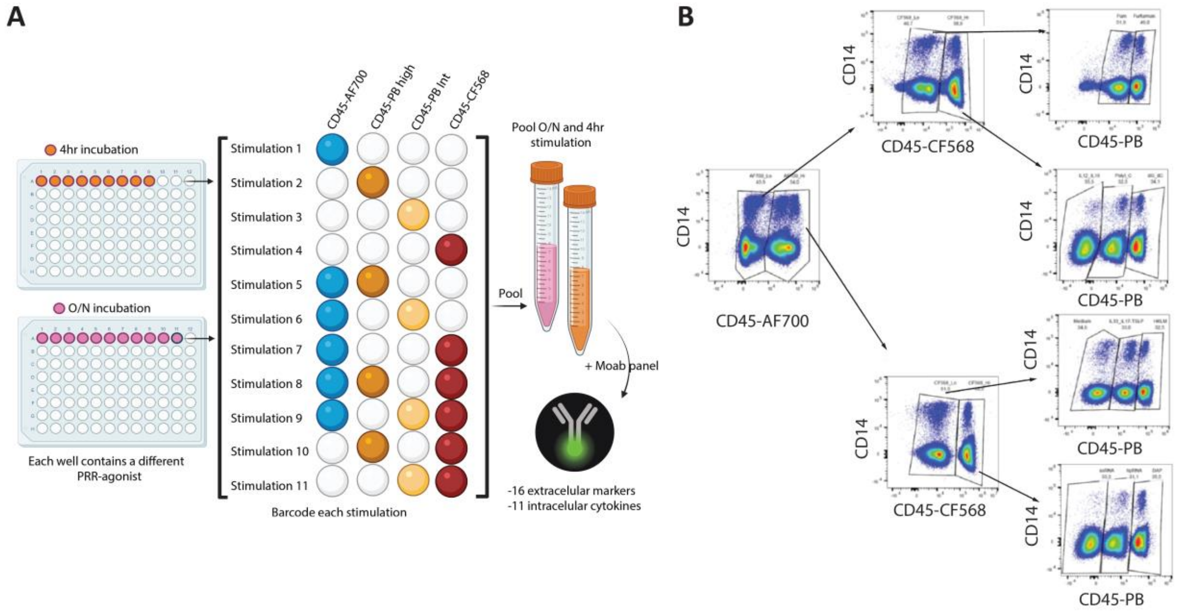

Supplementary Figure 1. Barcoding of PRR-agonist stimulated immune cells. A) Peripheral blood mononuclear cells (PBMCs) from each donor were split over 9 wells for 4 hour stimulation (orange) and 11 wells for overnight stimulation (pink) to stimulate with 18 different PRR-agonists and 2 medium controls. After stimulation, each well from each plate (4 hours or overnight) was barcoded according to the schematic using CD45-AF700, CD45-PB^{high}, CD45-PB^{int} and CD45-CF568. Barcoded wells were then pooled and stained using monoclonal antibodies for extracellular markers and after fixation and permeabilization with intracellular markers. Created in BioRender. *Jochems, S. (2025) https://BioRender.com/qgki19e* **B**) After acquisition of the pooled and barcoded samples (4hours and overnight), each barcoded stimulation was manually gated and exported. Shown is a representative pooled sample containing overnight stimulated conditions.

Abbreviations; PRR, pattern recognition receptor; AF700, AlexaFluor700; PB, PacificBlue; CF568, Cyanine-based Fluorescent 568

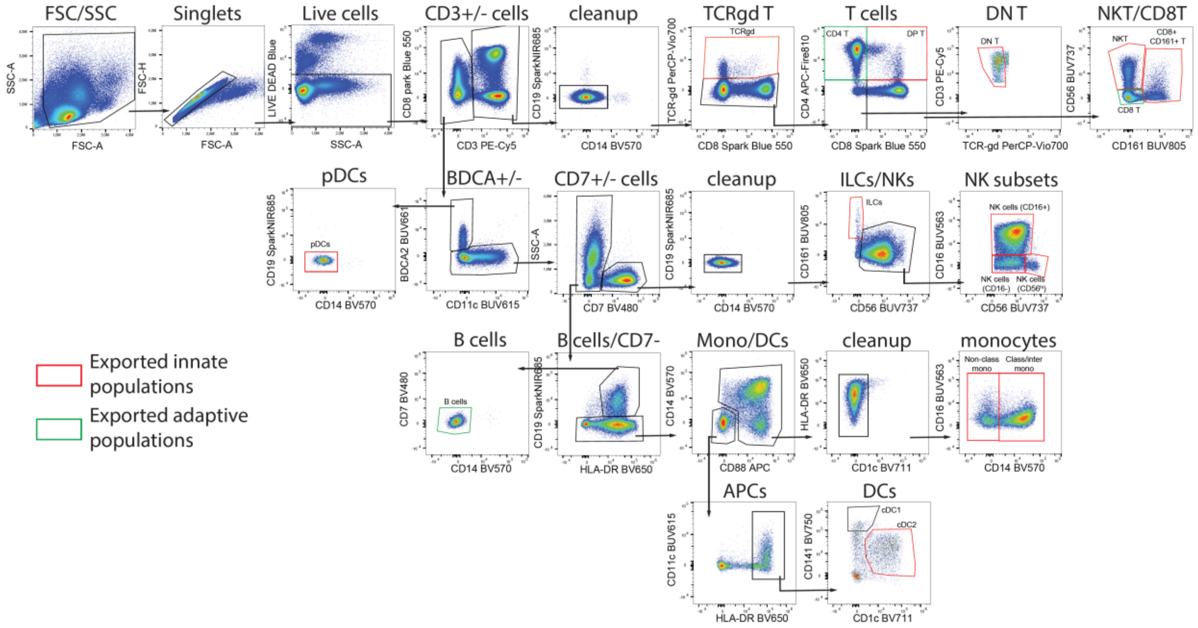

Supplementary Figure 2. Manual gating and exporting of immune cell populations after stimulation. For gating innate and adaptive immune cell populations after stimulation, Peripheral Blood Mononuclear Cells (PBMCs) were thawed, stimulated with 18 PRR-agonists, barcoded using different CD45 monoclonal antibodies and stained with a cocktail of antibodies targeting extracellular proteins. Single-cell live lymphocytes were first gated before the CD3+ lymphocytes were separated. From the CD3- subset pDCs were gated and CD3-CD7+ lymphocytes were separated from the myeloid lineage. Innate (red gate) and adaptive (green gate) immune cell populations were exported for further analyses. Intermediate (CD14+CD16+) and classical (CD14+CD16-) monoyctes were exported as one mixed population due to downregulation of CD16 after stimulation.

Abbreviations; ILCs, Innate lymphoid cells; DP T, double-positive T cells ($CD4^{+}CD8^{+}$); DN T, double-negative T cells ($CD4^{-}CD8^{-}$); mono, monocytes; pDC, plasmacytoid dendritic cells; cDC, classical dendritic cells; NK cells, natural killer cells; NKT cells, natural killer T cells; APC, Antigen presenting cells; BDCA2, blood dendritic cell antigen 2

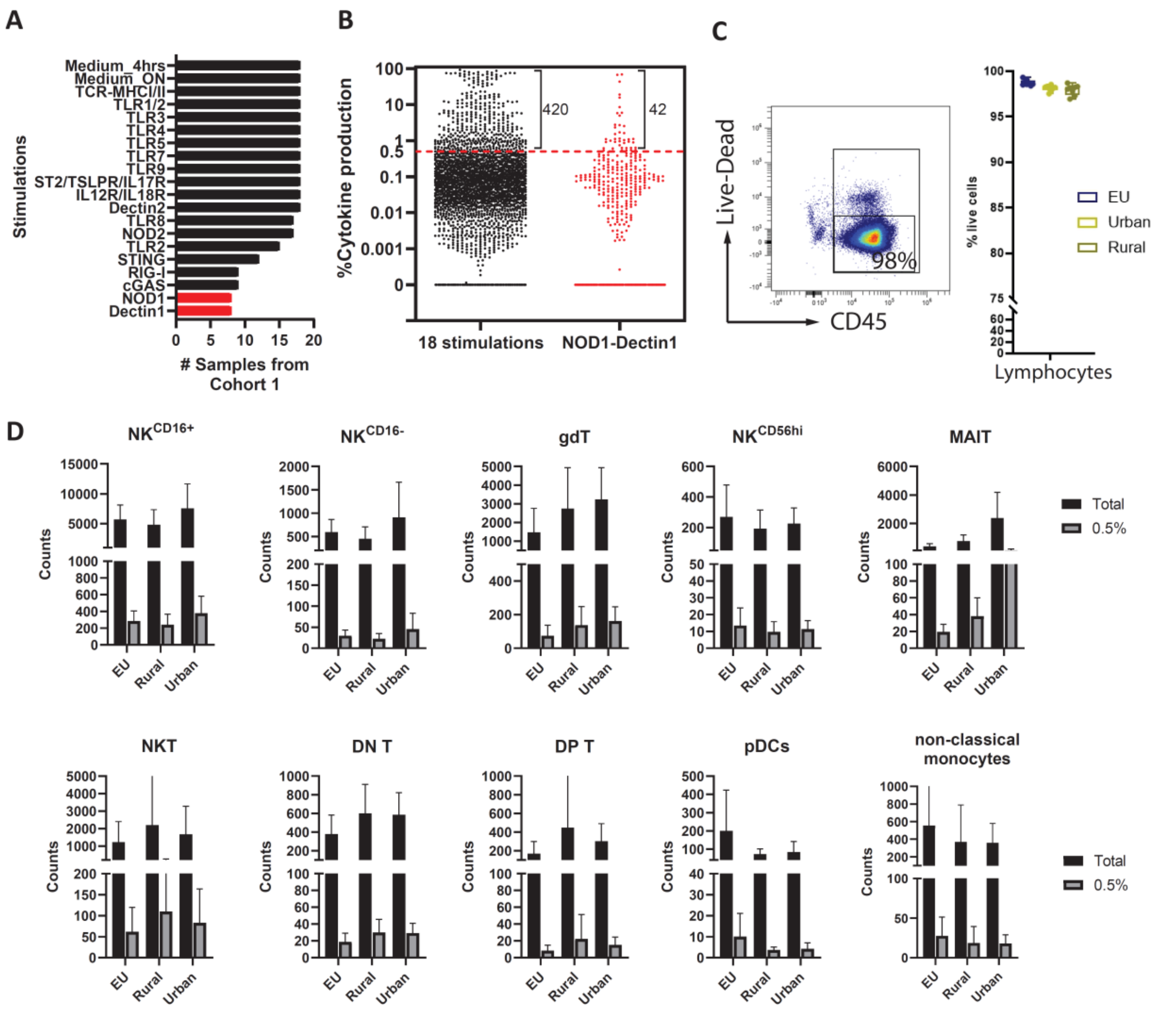

Supplementary Figure 3. Quality control of samples that were stimulated per PRR agonist. **A**) Shown are the number of samples for each stimulation of cohort 1. For comparisons between groups a cut-off of at least 9 samples was selected. Stimulations in red indicate stimulation that did not fulfil this criteria. **B**) Dotplots whereby each dot represents the average cytokine production per cytokine (n=11) of each population (n=13) for every stimulation. In total 462 conditions showed more than 0.5% cytokine production. Stimulations for NOD1 and Dectin1 were dropped when differences between groups of cohort 1 were compared due to limited numbers of samples. **C**) Shown is the viability of lymphocytes directly after thawing for representative samples from EU, urban and rural Indonesians. **D**) Average number of cells that were recorded for the medium controls (4 hours and overnight) for each population per group. The lower-limit of counts for 0.5% is shown that was used as cut-off.

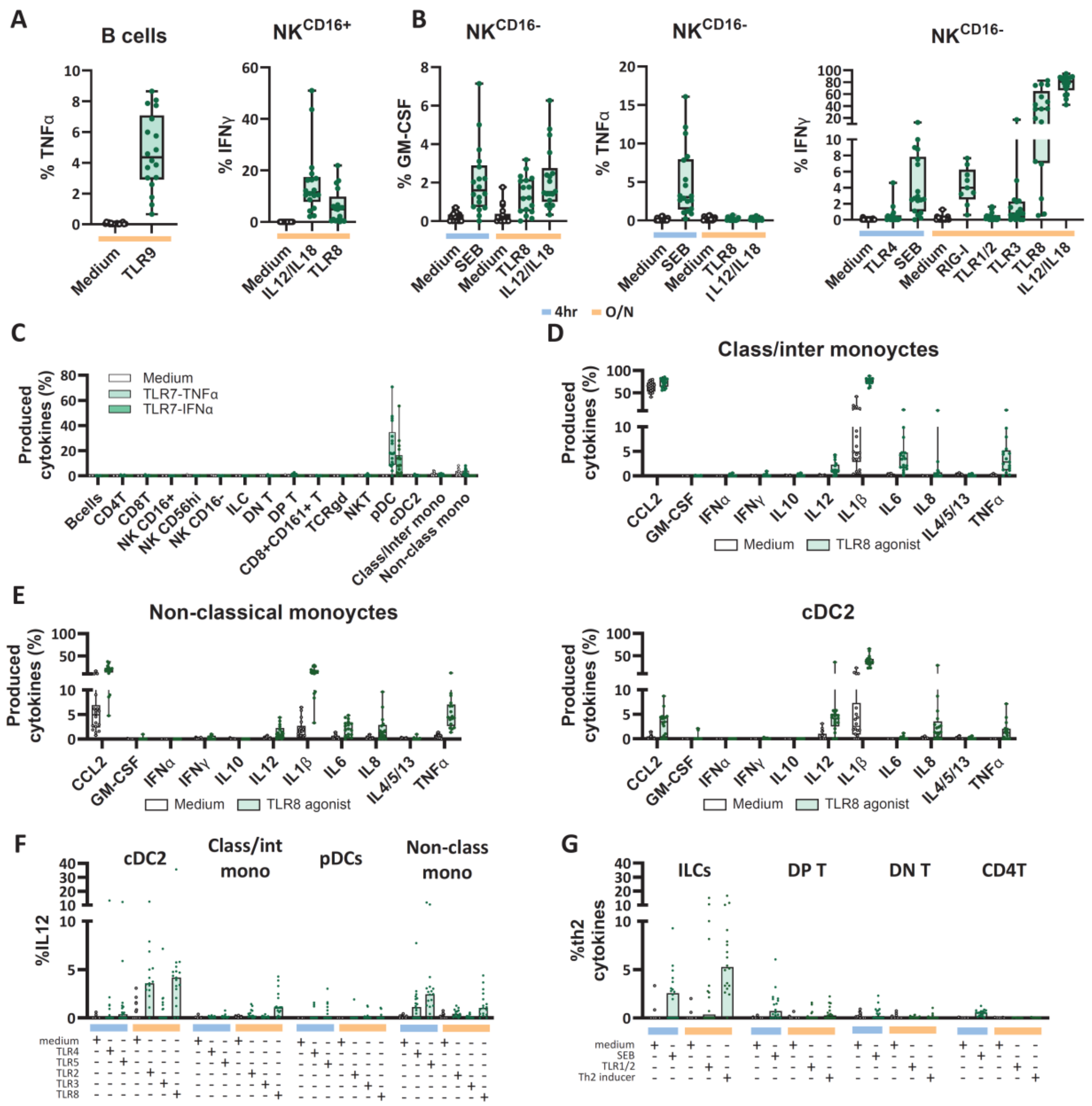

Supplementary Figure 4. Innate and adaptive immune cell populations and their function against different pattern recognition receptor agonists. To study the response of innate populations to stimulation with different PRR-agonists, 18 peripheral mononuclear cell (PBMC) samples (cohort 1) were used to generate a holistic overview of the various PRR signalling pathways across the immune system. **A and B**) Shown are boxplots of populations that only respond to TLR8 and TLR9 stimulation (**A**) and populations that respond to multiple PRR agonists and produce different cytokines (**B**). Stimuli are coloured below the x-axis according to stimulation duration (blue; 4 hours, orange; overnight). **C,D and E**) Shown are boxplots of populations that solely respond to TLR7 stimuli (**C**) and different subsets of monocytes that produce multiple different cytokines in response to TLR8 stimulation (**D and E**). **F and G**). Boxplots showing which populations are responsible for IL12 production (**F**) or production of th2-cytokines (G) in response to different PRR agonists.

Abbreviations; TLR, Toll-Like Receptor; ILCs, Innate lymphoid cells; DP T, double-positive T cells ($CD4^{+}CD8^{+}$); DN T, double-negative T cells ($CD4^{-}CD8^{-}$); mono, monocytes; pDC, plasmacytoid dendritic cells; cDC, classical dendritic cells; NK cells, natural killer cells.

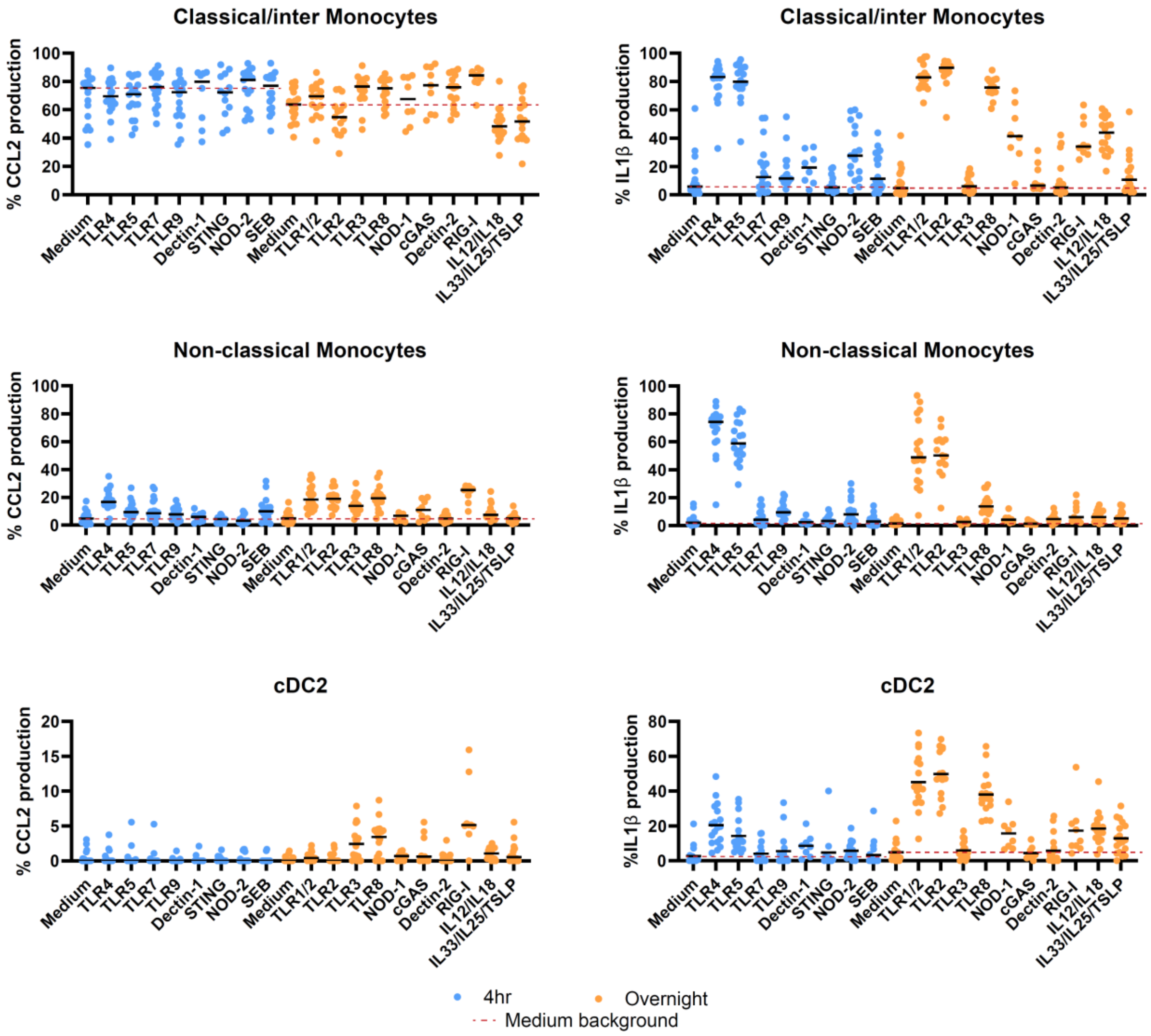

Supplementary Figure 5. Spontaneous release of CCL2 and IL1β by monocytes and cDCs. The IL1β and CCL2 production of monocytes and cDC2s from 18 peripheral mononuclear cell (PBMC) samples from cohort 1 are depicted in response to different PRR-agonists. Stimuli are coloured according stimulation duration (blue; 4 hours, orange; overnight). Red dotted lines indicate medium background for 4 hour and overnight stimuli separately.

Abbreviations;TLR, Toll-Like Receptor; cDC, classical dendritic cells; NK cells,

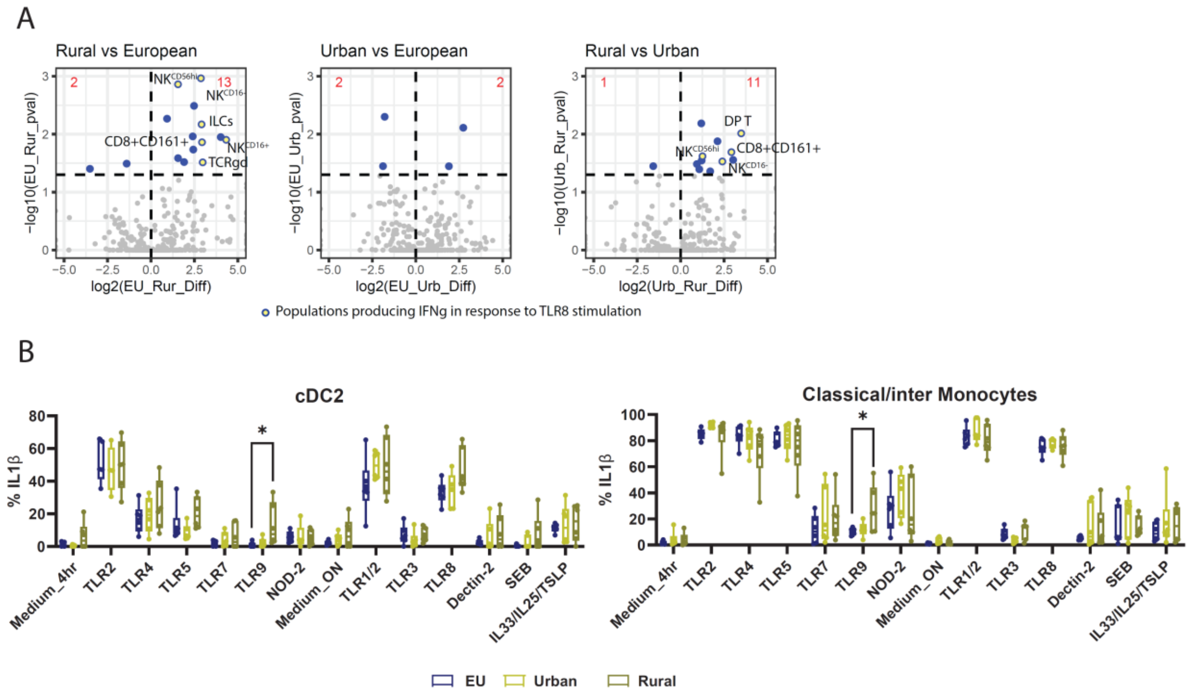

Supplementary Figure 6. Functional differences of immune cell populations between people from different geographical areas. A) Volcano plots of 420 cytokine producing (>0.5%) immune cell populations in response to different PRR agonists between different study groups. Populations that produce IFNγ in response to TLR8 stimulation are highlighted by yellow filled circles. **B)** Boxplots of production of IL1β of cDC2s and classical/intermediate monoyctes in response to different PRR-agonists.

Statistical differences were assessed with a Limma test with correction for testing multiple groups using Bonferroni correction. **P* < 0.05; ***P* < 0.01; ****P* < 0.001. Floating box plots depict median with min-max

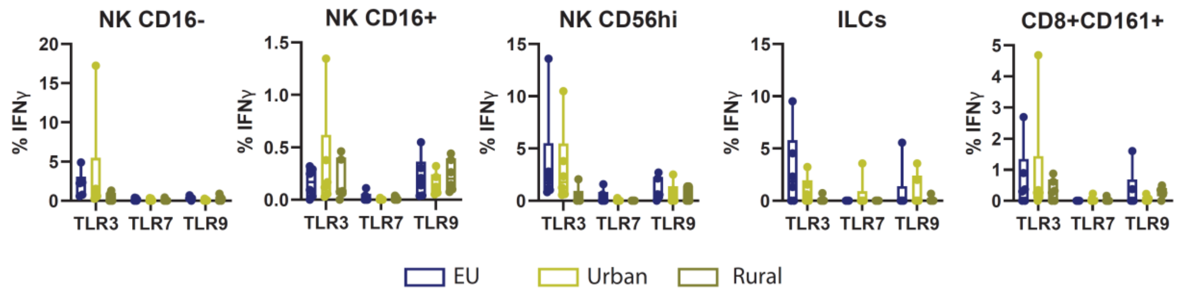

Supplementary Figure 7. Functional response to endosomal TLR agonists by innate immune cell populations. Boxplots of production of IFNγ of innate lymphoid-derived immune cell populations (cohort 1, n=6 per group) in response to different endosomal PRR-agonists.

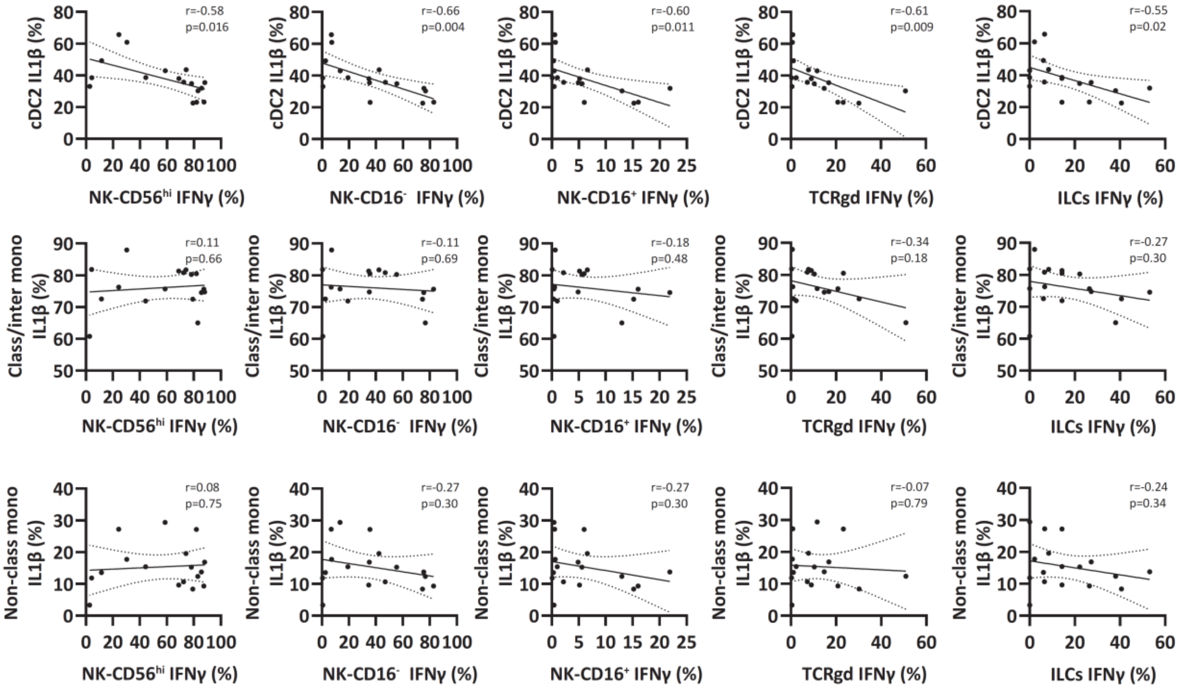

Supplementary Figure 8. Correlation plots of TLR8-agonist responding myeloid and lymphoid-derived innate populations. Correlation plots are shown for IFNγ producing lymphoid-derived innate populations against IL1β producing cDC2s, (top row) IL1β producing class/intermediate monocytes (middle row) and IL1β producing non-class monocytes (bottom row) from cohort 1 (n=6 per group). Spearman correlation coefficients (r) and P values for each comparison are shown within each plot.

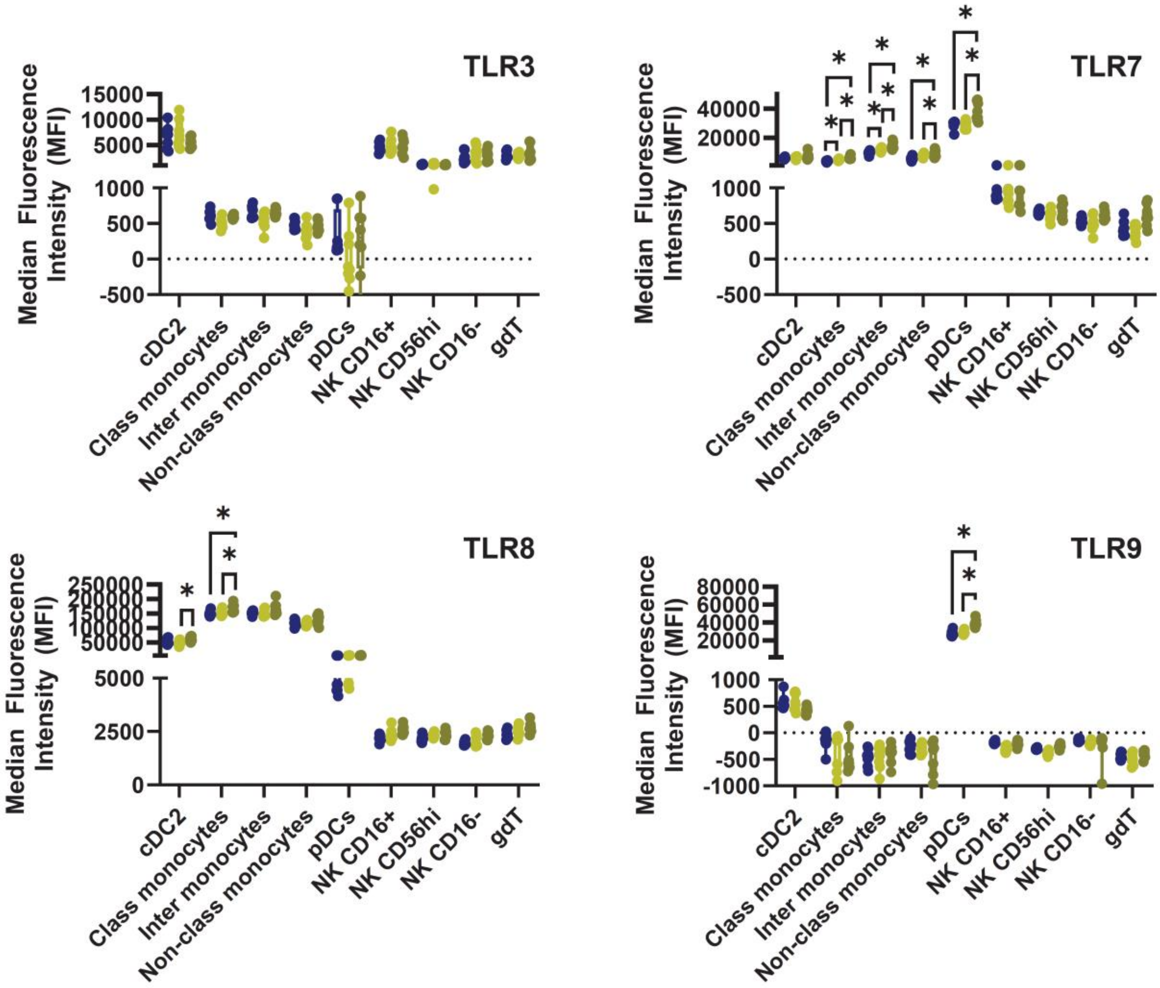

Supplementary Figure 9. TLR-expression of innate immune cell populations. PBMCs from cohort 3 (n=8 per group) were used to measure the TLR expression of different lymphoid-derived innate immune cell populations and myeloid-derived innate immune cell populations. Median Fluorescence Intensity (MFI) of endosomal TLRs (TLR3, TLR7, TLR8 and TLR9) is shown for different innate immune cell population.

Statistical differences were assessed with One-way Anova with FDR correction for multiple testing. **P* < 0.05; ***P* < 0.01; ****P* < 0.001. Floating box plots depict median with min-max

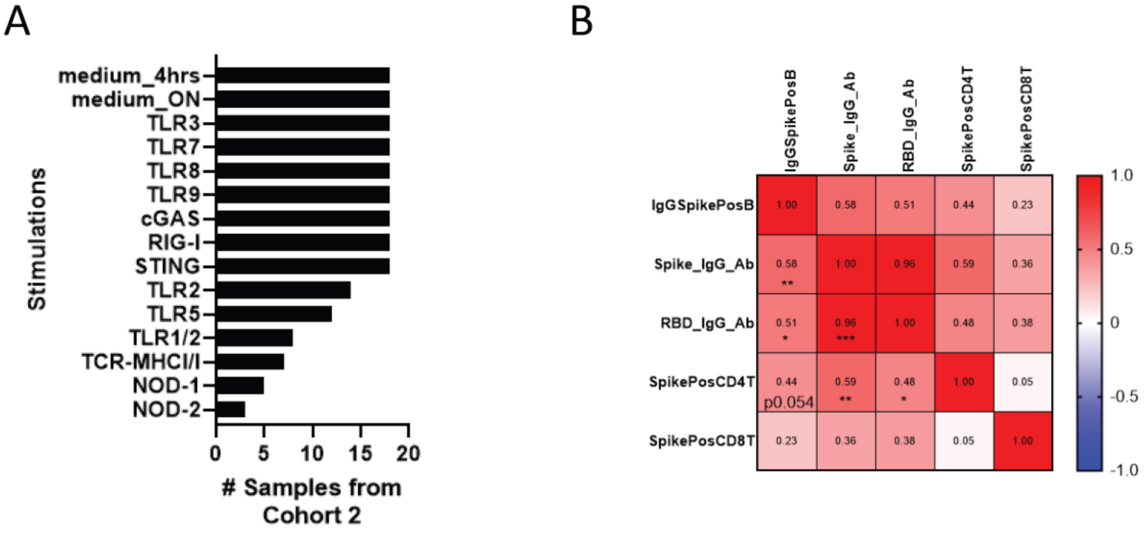

Supplementary Figure 10. Overview of stimulations from cohort 2 and correlation matrix of vaccine response. A) Shown are the number of samples for each stimulation of cohort 2 (n=18 PBMC samples). Priority was given for endosomal sensors. All samples could be stimulated the endosomal sensors (TLR3,7,8 9, and cGAS, RIG-1 and STING). **B)** Correlation heatmap reporting Spearman correlation coefficients (r) as color and number and P values as asterix for each comparison.

Statistical differences were assessed with Spearman correlations (B). *$P < 0.05$; **$P < 0.01$; ***$P < 0.001$; ****$P < 0.0001$.

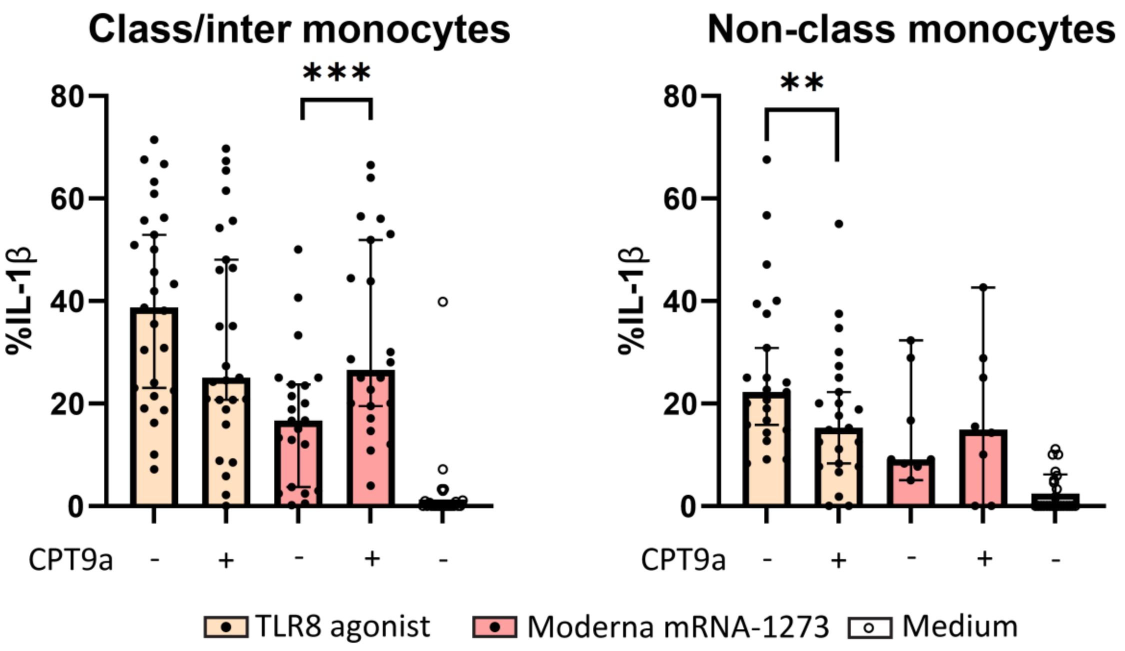

Supplementary Figure 11. Response of myeloid-derived innate populations to moderna mRNA-1273. PBMCs from cohort 1 were stimulated with TLR8 agonist ssRNA40 and Moderna mRNA1273 with and without TLR8 inhibitor CPT9a. Production of IL1β was measured for classical/intermediate monocytes and non-classical monocytes.

Statistical differences were assessed with Wilcoxon signed-rank tests. *P < 0.05; **P < 0.01; ***P < 0.001; ****P < 0.0001. Bar plots depict median with 95%CI

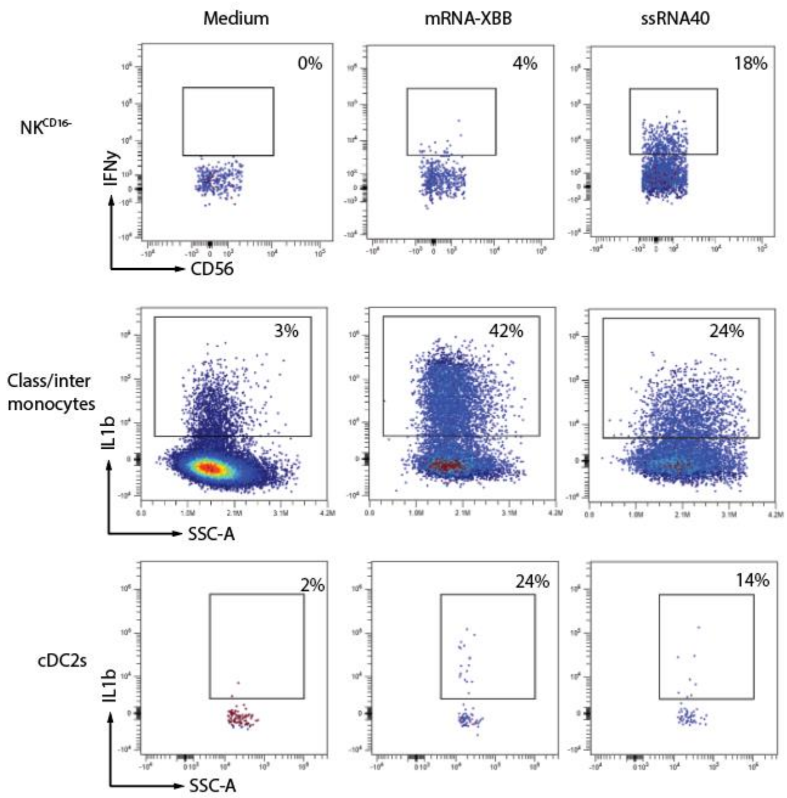

Supplementary Figure 12. Representative example of mRNA-XBB responding lymphoid and myeloid-derived innate cells. Flow cytometry plots are shown from a representative PBMC sample from cohort 4 stimulated with mRNA-XBB or ssRNA40. Gating was set for medium control and applied to all samples and conditions.
